# On the little-known genus *Meilichius* with descriptions of three new species from China (Coleoptera, Endomychidae)

**DOI:** 10.3897/zookeys.1276.181983

**Published:** 2026-04-08

**Authors:** Ling-Xiao Chang, Pan-Pan Li, Ming Bai, Wen-Xuan Bi

**Affiliations:** 1 Department of Life Sciences, Natural History Museum of China, Beijing 100050, China College of Agriculture, Guangxi University Nanning China; 2 State Key Laboratory of Animal Biodiversity Conservation and Integrated Pest Management, Institute of Zoology, Chinese Academy of Sciences, Beijing 100101, China Institute of Zoology, Chinese Academy of Sciences Beijing China; 3 Guangxi Key Laboratory of Agro-environment and Agric-products Safety, National Demonstration Center for Experimental Plant Science Education, College of Agriculture, Guangxi University, Guangxi, Nanning 530004, China Department of Life Sciences, Natural History Museum of China Beijing China; 4 Room 401, No. 2, Lane 155, Lianhua South Road, Shanghai, 201100, China Unaffiliated Shanghai China

**Keywords:** Asia, biodiversity, Coccinelloidea, Cyclotominae, handsome fungus, new species, Oriental Region beetles, taxonomy

## Abstract

This study is based on a comprehensive morphological examination of physical specimens, high-resolution photographs, and primary literature covering all known species of the genus *Meilichius* Gerstaecker. Building upon this examination and utilizing specimens collected from China, three new species of *Meilichius* are described: *M. chebalingensis* Chang & Li, **sp. nov**., *M. speciosus* Chang & Bi, **sp. nov**. and *M. tomaszewskae* Chang & Bi, **sp. nov**. Illustrations and diagnostic characters of the new species are presented, with a key to all known species of *Meilichius*. In addition, this study presents the first mitochondrial COI barcode data and ecological information for the recently described species *M. wukong* Wang & Tomaszewska.

## Introduction

The genus *Meilichius* was established by Gerstaecker (1857), with *M. nigricollis* from Malaysia by monotypy. Subsequently, Gemminger and Harold (1876) misspelled the generic name as *Milichius*, an incorrect subsequent spelling of *Meilichius*. In 1920, Arrow synonymized *Thelgetrum* Gorham, 1875 with *Meilichius* and also placed Gibbiger Csiki, 1900 into synonymy under the latter, arguing that no stable diagnostic characters could distinguish it from *Meilichius*. Later, Strohecker (1944) described one new species, *M. aeneoniger* under *Meilichius* and advocated for the retention of Gerstaecker’s original spelling based on the principle of priority.

*Meilichius* was previously classified within the endomychid subfamily Endomychinae (e.g., Strohecker 1953; Tomaszewska 2000). However, evidence from the molecular study by Robertson et al. (2015) recovered this subfamily as non-monophyletic. Given the strong support for the polyphyly of Endomychinae, the composition of Cyclotominae was redefined by Robertson et al. (2015), and *Meilichius* with *Bolbomorphus* Gorham, 1887, *Cyclotoma* Mulsant, 1851, and *Eucteanus* Gerstaecker, 1857 were classified in the subfamily Cyclotominae.

Recently, Wang and Tomaszewska (2025) described *Meilichius wukong* Wang & Tomaszewska, 2025 from Guangxi, China. In the present study, we have also collected numerous specimens of this species, expanding its known distribution to include both Guangxi and Guangdong, China. Prior to this research, apart from *M. wukong*, the genus *Meilichius* comprised 20 recognized species, only three of which were recorded from China: *M. erotyloides*Strohecker, 1951 (Hainan), *M. klapperichi* Mader, 1941 (Fujian, Taiwan), and *M. multimaculatus* Sasaji, 1970 (Taiwan) (Shockley et al. 2009). During our examination of Chinese Endomychidae material, three additional new species of *Meilichius* were identified and are formally described here.

## Materiasl and methods

Type specimens of the new species described herein are deposited in the following institutions and private collections:

**CBWX** Collection of Wen-Xuan Bi, Shanghai, China;

**NHMC** National Natural History Museum of China, Beijing, China;

**IZCAS** Institute of Zoology, Chinese Academy of Sciences, Beijing, China.

The examined specimens of known species are deposited in the following institutions:

**CAS** California Academy of Sciences;

**NHMUK** Natural History Museum, London, United Kingdom;

**USNM** United States National Museum;

**ZFMK** Zoologisches Forschungsmuseum Alexander Koenig.

The specimens were examined, dissected, and measured using an Olympus SZX10 dissecting microscope. Measurements and abbreviations used in the descriptions are as follows: body length (**BL**): length between the apex of the clypeus and the elytral apex along the midline; body height (**BH**): maximum height of the body; pronotum length (**PL**): length of the pronotum along the mid-line; pronotum width (**PW**): maximum width of the pronotum; elytra length (**EL**): length from anterior margin to apices of the elytra; elytra width (**EW**): widest part of elytra. Morphological terminology follows Tomaszewska (2005) and Yoshitomi (2020).

Habitus images and morphological details were captured using a Canon EOS 5D Mark III camera equipped with a Canon MP-E 65 mm f/2.8 1–5× macro lens and a Canon MT-24EX Macro Twin Lite flash. Images of the male aedeagus and genital segment were taken with a Canon EOS 5D camera equipped with a M Plan Apo 5× objective and a WeMacro M26-to-M42 tube lens, using two Canon 430EXIII-RT flashes as the light source. All figures were processed and composited in Adobe Photoshop 2023.

One specimen of *Meilichius wukong* was preserved in absolute ethanol and submitted to Qingke Biotechnology Co., Ltd. (Beijing, China) for molecular analysis. The company carried out total genomic DNA extraction, PCR amplification, and subsequent Sanger sequencing following standard procedures. The mitochondrial COI gene was amplified using the universal primer pair LCO1490 (5' GGTCAACAAATCATAAAGATATTGG-3') and HCO2198 (5' TAAACTTCAGGGTGACCAAAAAATCA-3') (Folmer et al. 1994). PCR products were purified and sequenced by the same provider. The resulting sequence was deposited in the GenBank database under the accession number PX637245.

Genomic DNA was extracted from fungal fruiting bodies following this protocol: small fragments of fungal fruiting bodies were frozen in liquid nitrogen and ground into a fine powder using a mortar and pestle. Genomic DNA was extracted using the TGuide S16 automated nucleic acid purification system (OSE-S16) in combination with the TGuide Smart Plant Genomic DNA Kit (Tiangen Biotech Co., Ltd., Beijing, China), following the manufacturer’s instructions. The ITS region was amplified using the primers ITS5 (5'-GGAAGTAAAAGTCGTAACAAGG-3') and ITS4 (5'-TCCTCCGCTTATTGATATGC-3'). PCR reactions were performed in a 30 μL volume containing 15 μL of 2× Taq Mix (Tiangen), 1 μL of each primer (10 μM), 1 μL of template DNA, and 12 μL of ddH_2_O. The thermal cycling conditions were as follows: initial denaturation at 95 °C for 3 min; 34 cycles of denaturation at 94 °C for 40 s, annealing at 57.2 °C for 45 s, and extension at 72 °C for 1 min; and a final extension at 72 °C for 10 min. PCR products were examined by electrophoresis on 1% agarose gels. Samples that produced clear bands were selected and subjected to Sanger sequencing at Beijing Tsingke Biotechnology Co., Ltd. The resulting sequences were edited and compared against the NCBI database using BLAST for identification.

### Key to genera of Cyclotominae

Modified from Strohecker 1953 and Tomaszewska 2000.

**Table:** 

1	Body orbicular; antennal club equal in length to remaining antennomeres	*** Cyclotoma ***
–	Body broadly oval; antennal club shorter than remaining antennomeres	**2**
2	Antennal club narrow, slightly flattened; scutellar shield triangular	*** Meilichius ***
–	Antennal club broad, flat; scutellar shield oval	**3**
3	Mandible with apex produced, chisel-shaped; mesosternum with intercoxal process longer than wide or nearly as long as wide	*** Eucteanus ***
—	Mandible molariform, apex very short; mesosternum with intercoxal process wider than long	*** Bolbomorphus ***

## Taxonomy

### Meilichius

Taxon classificationAnimaliaColeopteraEndomychidae

Gerstaecker, 1857

B096C62A-40FF-5284-8ABB-52ACB4CBDB51

Meilichius Gerstaecker, 1857: 240. Type species: Meilichius nigricollis Gerstaecker, 1857.
Thelgetrum Gorham, 1875: 314. Type species: Thelgetrum ampliatum Gorham, 1875.
Milichius Gemminger & Harold, 1876: 3737.
Gibbiger Csiki, 1900: 375. Type species: Milichius fasciatus Heller, 1898.

#### Diagnosis.

Species of *Meilichius* appear to be closely related to *Bolbomorphus* and *Cyclotoma*. However, *Meilichius* can be distinguished from *Bolbomorphus* by its smaller body size, ~ 3.0–6.0 mm in length (vs distinctly larger, ~ 7.0–10.0 mm); scutellar shield subtriangular (vs transversely oval). It differs from *Cyclotoma* in having the body broadly oval (vs orbicular); antennal club shorter than the remaining segments (vs subequal in length to the remaining segments).

##### New species descriptions

### Meilichius chebalingensis

Taxon classificationAnimaliaColeopteraEndomychidae

Chang & Li sp. nov.

E71D33AF-E428-55A3-B3F2-E6023F7A9648

https://zoobank.org/0F5445C0-7F34-4A3E-9D1F-F7025B05251A

Figs 1, 2

#### Type material.

***Holotype***: China • ♀; **Guangdong**, Shixin County (始新县), Chebaling (车八岭), 114.186785°N, 24.735523°E; 788 m; 1. IV. 2022; Pan-Pan Li leg.; (IZCAS); ***Paratypes***: China • 1♀; same collecting data as holotype; (NHMC).

#### Diagnosis.

*Meilichius chebalingensis* sp. nov. resembles *M. ampliatus*, *M. apicicornis*, *M. biplagiatus*, *M. brevicollis*, *M. callosus*, *M. fuscipes*, *M. nigricollis*, *M. pachycerus*, and *M. politus* in having elytra lacking metallic luster and maculae. However, *M. chebalingensis* can be distinguished as follows:

From *M. ampliatus* (Fig. 13): antennae almost uniformly brown to dark brown, without obvious color differentiation (vs antennomeres 1–2 and the terminal one brownish-yellow, antennomeres 3–10 brown to dark brown); elytra widest before the mid-length (vs distinctly behind the mid-length); elytral surface with coarse punctures (vs fine punctures); humeri weakly prominent (vs strongly prominent); legs and dorsal surfaces of different colors (vs concolorous).

From *M. apicicornis* (Fig. 14): antennae almost uniformly brown to dark brown (vs antennomeres 1–4 and the terminal one brown, antennomeres 5–10 black); elytra widest before the mid-length (vs near the mid-length); humeri weakly prominent (vs moderately prominent); legs and dorsal surfaces of different colors (vs of the same color).

From *M. biplagiatus* (Fig. 15): antennae and legs similar in color, differing from the dorsal surface (vs antennae, legs, and dorsal surface all similar in color); elytra widest before the middle (vs at the middle); elytral surface with coarse punctures (vs rather fine punctures); humeri weakly prominent (vs strongly prominent).

From *M. brevicollis* (Fig. 16): antennae almost uniformly brown to dark brown (vs antennomeres 1–4 brown, antennomeres 5–10 and the basal half of the terminal one black); elytra widest before the mid-length (vs at the mid-length); elytral surface with coarse punctures (vs rather fine punctures); legs and dorsal surfaces of different colors (vs of the same color).

From *M. callosus* (Fig. 17): antennae almost uniformly brown to dark brown (vs antennomeres 1–7 and the terminal one brown, antennomeres 8–10 black); elytra widest before the mid-length (vs near the mid-length); humeri weakly prominent (vs strongly prominent, with orange raised areas); legs and dorsal surfaces of different colors (vs of the same color).

From *M. fuscipes* (Fig. 20): terminal antennomere mostly dark brown (vs orange); elytral surface with coarse punctures (vs fine punctures); humeri weakly prominent (vs strongly prominent).

From *M. pachycerus* (Fig. 28): antennae almost uniformly brown to dark brown (vs antennomeres 1–5 ferruginous, 6–8 and 11 black, 9–10 pale yellow); elytra widest before the mid-length (vs near the mid-length); legs and dorsal surfaces of different colors (vs of the same color).

From *M. politus* (Fig. 29): antennae almost uniformly brown to dark brown (vs antennomeres 1–4 brownish-yellow, 5–11 dark brown); elytra widest before the middle (vs near the middle); elytral surface with coarse punctures (vs extremely fine punctures); legs and dorsal surfaces of different colors (vs of the same color).

#### Description.

**Male**. Unknown.

**Figure 1.**
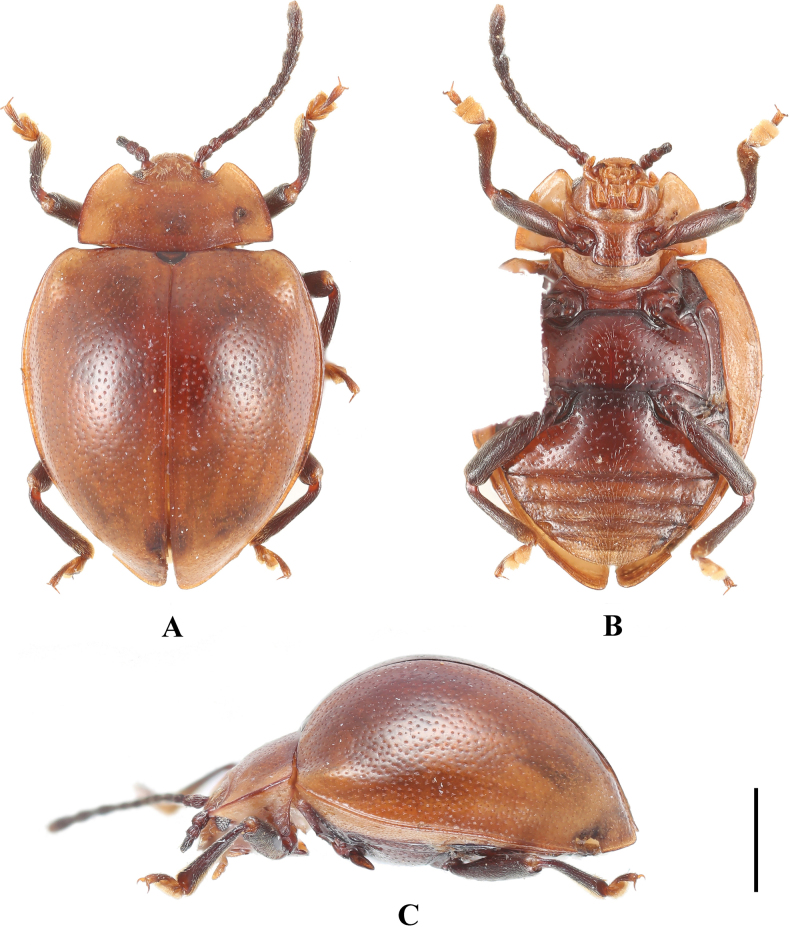
Habitus of *Meilichius chebalingensis* sp. nov. **A**. Dorsal view; **B**. Ventral view; **C**. Lateral view. Scale bar: 1.0 mm.

**Female. *Body*** (Fig. 1) oval, convex, surfaces smooth, rather shiny. Eyes black; head, pronotum and elytra pale brown; antennae and legs brown except apical 1/3 of terminal antennomeres and tarsi, both of which pale brown; ventral surfaces mostly dark brown to brown.

***Head***. Antenna 11-segmented with scape rather long and stout, 3.0× longer than pedicel; pedicel slightly longer than wide; antennomere 3 ~ 2.0× longer than antennomere 2 and nearly as long as antennomere 4; antennomere 4 slightly longer than antennomere 5; antennomeres 6–8 distinctly longer than wide and gradually shorter; club composed of three antennomeres, narrow and loosely articulated; terminal antennomeres 3.0× as long as wide. Maxilla with palp comparatively stout; terminal palpomere 1.5× as long as wide, sides converging toward apex, obtusely rounded apically.

***Thorax***. Pronotum with anterior margins very narrowly bordered, lateral margins moderately bordered; disc weakly convex, median furrow absent. Pronotal surface polished between punctures, punctation rather dense and rather coarse; lateral sulci distinct, linear, extending to 1/4 of pronotal length; basal sulcus shallow and weakly curved. Prosternal process widely separates front coxae, ~ 2.0× as wide as longest coxal diameter and narrower than intercoxal process of mesoventrite, widening behind front coxae; sides curved outward, weakly curved at apex. Elytral sides strongly curved, widest beyond mid-length, posterior 1/3 abruptly converged and slightly produced at apex; elytral epipleuron broad, abruptly tapered beyond mid-length and obsolete at apex. Humeri weakly prominent; elytral surface polished between punctures, punctation as large as pronotal ones, dense and coarse.

***Abdomen***. Ventrite 1 almost as long as three subsequent ventrites combined; ventrites 2–4 gradually shorter in length. Ventrite 5 with posterior margin rounded.

***Measurements* (in mm)**. BL 4.1–4.3, BH 2.0–2.1, PL 0.9–1.0, PW 1.8–1.9, EL 3.3–3.5, EW 2.7–2.8, BH/BL 0.5, PL/PW 0.5, EL/EW 1.2–1.3, EL/PL 2.6–3.0, EW/PW 1.5.

#### Etymology.

The species is named after its type locality, Chebaling National Nature Reserve, Guangdong Province, China; adjective.

#### Distribution.

China: Guangdong.

#### Ecology.

**Figure 2.**
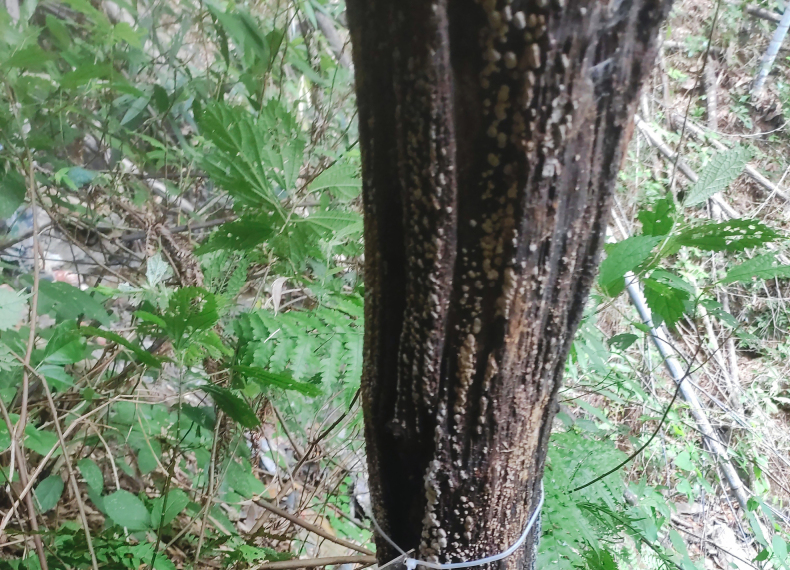
*Xylobolus austrosinensis* growing on a dead tree serves as an attractant for *Meilichius chebalingensis* sp. nov.

The holotype and paratype of *Meilichius chebalingensis* sp. nov. were collected using Mycophagy insects windows trap with a wood-decaying fungus growing on a tree (Fig. 2). The fungus was subsequently identified as *Xylobolus austrosinensis* Ji & Zhou, 2018 based on Internal Transcribed Spacer (ITS) sequencing.

### Meilichius speciosus

Taxon classificationAnimaliaColeopteraEndomychidae

Chang & Bi sp. nov.

A3928DD9-F378-524B-B9ED-6857776FD778

https://zoobank.org/7A0B9679-64B4-45B0-83E1-49D337C760B3

Figs 3, 4, 5

#### Type material.

***Holotype***: China • ♂; **Fujian**, Shanghang County (上杭县), Meihuashan National Nature Reserve (梅花山保护区), Shuangche Village (双车村); 480 m; 6. XI. 2008; local collector leg.; (CBWX); ***Paratype***: China • 1♀; same collecting data as holotype; (CBWX).

#### Diagnosis.

*Meilichius speciosus* sp. nov. resembles *M. ornatus* in having each elytron with two large maculae of irregular shape, which occupy the basal and apical 1/3 of the elytral length. However, *M. speciosus* differs from *M. ornatus* in having the body oval (vs broadly oval); elytra widest near the mid-length (vs before the mid-length); dorsal surfaces of pronotum and elytra of different color (vs concolorous); elytra with copper-yellow luster (vs without luster).

#### Description.

**Figure 3.**
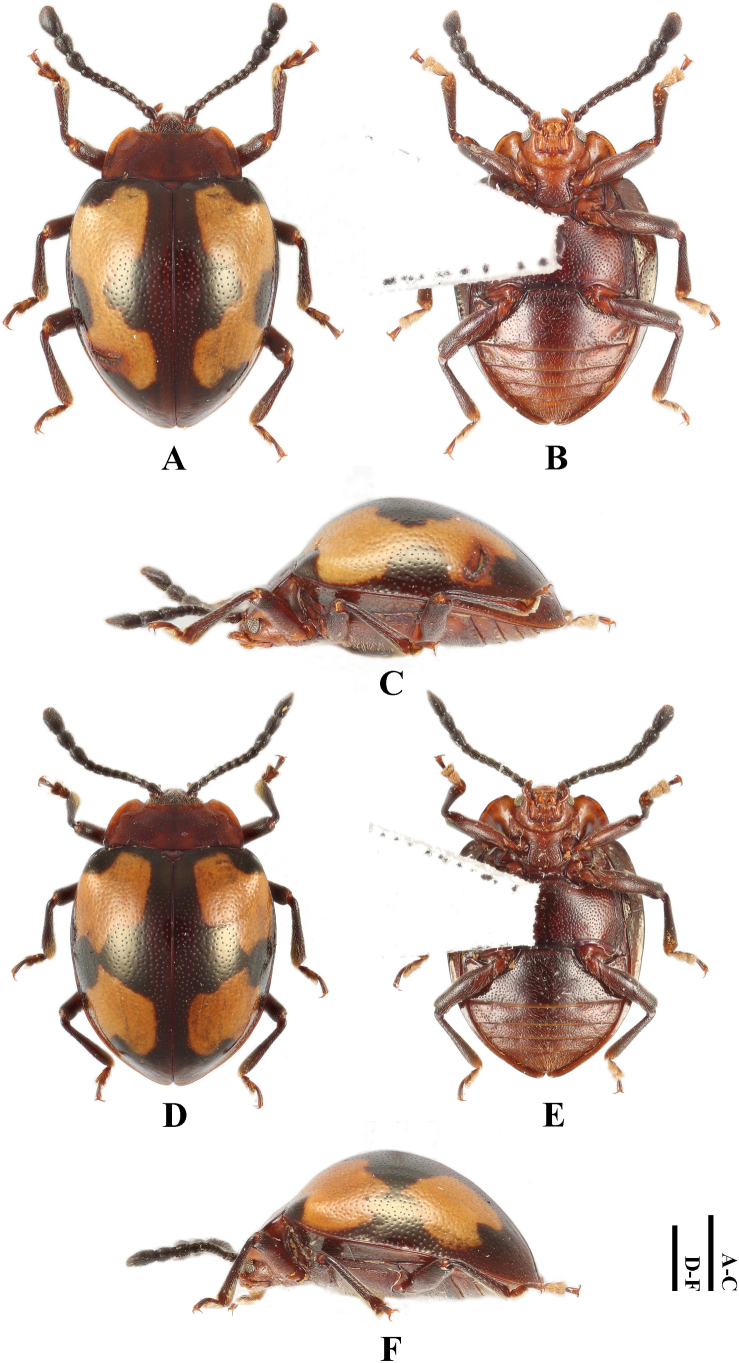
Habitus of *Meilichius speciosus* sp. nov. **A–C**. Male; **D–F**. Female: **A, D**. Dorsal view; **B, E**. Ventral view; **C, F**. Lateral view. Scale bars: 1.0 mm.

**Male. *Body*** (Fig. 3A–C) oval, convex, surfaces smooth, rather shiny. Eyes black; pronotum wine red; head, antennal scape, and legs red-brown; antenna except scape gradually from brown to black; elytra blackish red-brown with copper-yellow luster; each elytron with two large orange maculae; apical margin of the anterior macula and the basal margin of the posterior macula connected medially by a wide band. ventral surfaces mostly dark brown to brown; elytral epipleuron with copper-yellow luster.

***Head***. Antenna 11-segmented with scape rather long and stout, 3.0× longer than pedicel; pedicel slightly longer than wide; antennomere 3 ~ 2.0× longer than antennomere 2 and nearly as long as antennomere 4; antennomere 4 slightly longer than antennomere 5; antennomeres 6–8 longer than wide and approximately equal in length; club composed of three antennomeres, narrow and loosely articulated; terminal antennomeres twice as long as wide. Maxillary terminal palpomere 2.0× as long as wide, sides gradually tapering toward apex, obliquely truncate apically.

***Thorax***. Pronotum with anterior and lateral margins very narrowly bordered; disc weakly convex, median furrow absent. Pronotal surface polished between punctures, punctation rather dense and fine; lateral sulci distinct, linear, extending to 1/4 of pronotal length; basal sulcus shallow and weakly curved. Prosternal process widely separates front coxae, ~ 2.0× as wide as longest coxal diameter and narrower than intercoxal process of mesoventrite, widening behind front coxae; sides near mid-length strongly curved outward, rounded at apex. Elytral sides rounded, widest near mid-length, posterior 1/3 abruptly converged and slightly produced at apex. Anterior elytral maculae located before 1/2 of elytron, outer margin almost touching lateral margin of elytra, inner margin distant from elytral suture; anterior margin deeply emarginate enclosing humeri; posterior margin tapering toward middle, then produced backward as a band and connected with the anterior margin of posterior elytral maculae. Posterior elytral maculae located behind 1/2 of elytron, outer margin distant from lateral margin of elytra, inner margin distant from elytral suture. Elytral epipleuron rather broad, abruptly tapered beyond mid-length and obsolete at apex. Humeri weakly prominent; elytral surface polished between punctures, punctation dense, coarser than pronotal ones.

**Figure 4.**
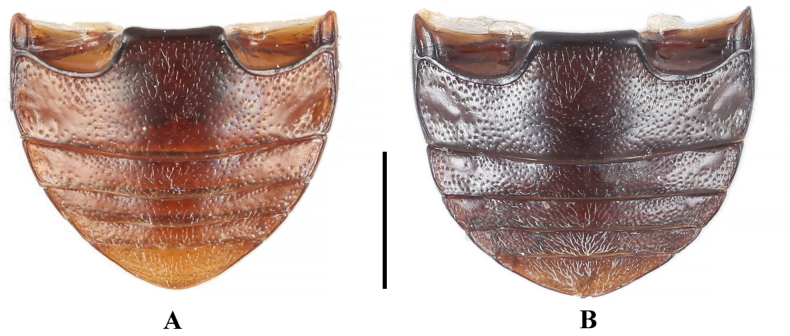
Abdomen of *Meilichius speciosus*. **A**. Male; **B**. Female. Scale bar: 1.0 mm.

**Figure 5.**
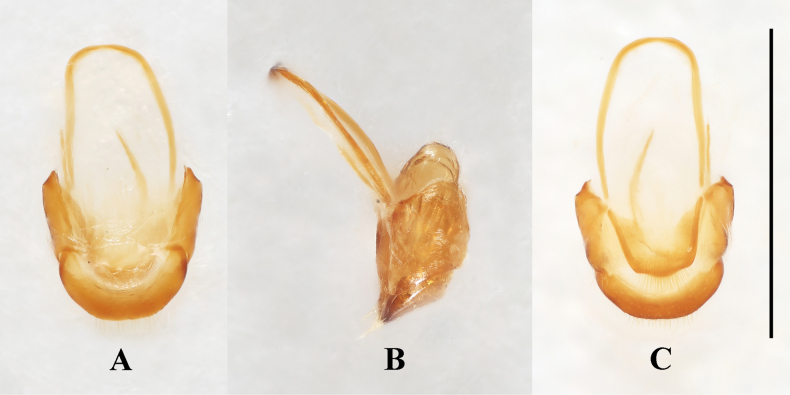
Male genital segment of *Meilichius speciosus* sp. nov. **A**. Dorsal view; **B**. Lateral view; **C**. Ventral view. Scale bar: 1.0 mm.

***Abdomen*** (Fig. 4). Ventrites 1–3 with smooth and distinctly non-punctate areas on each side; ventrite 1 slightly shorter than three subsequent ventrites combined; ventrites 2–4 gradually shorter in length. Ventrite 5 with posterior margin narrowly rounded at apex. Male genital segment (Fig. 5) with paired apophyses, narrow and fused into a U-shape; subgenital plate wide and tongue-shaped; sternite IX narrow, slightly convex, and setose at apex; tergite IX comprises two large laterotergites connected by a wide intervening membrane; tergite X subtruncate apically.

**Figure 6.**
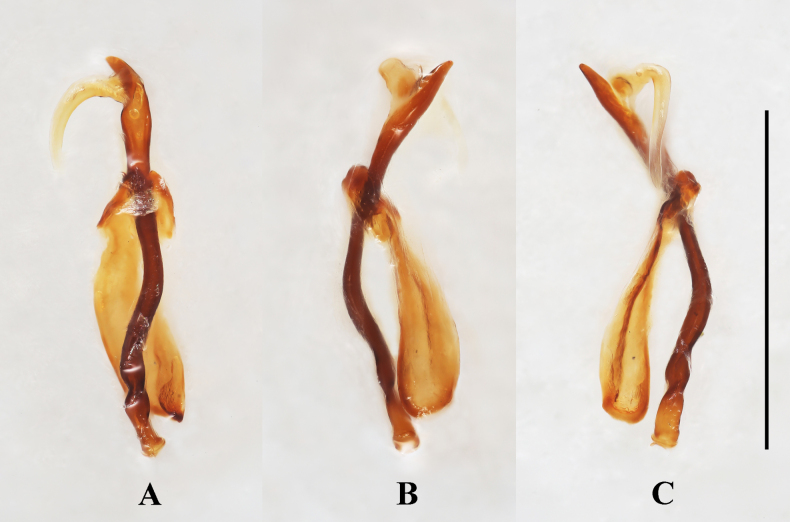
Aedeagus of *Meilichius speciosus* sp. nov. **A**. Ventral view; **B**. Left lateral view; **C**. Right lateral view. Scale bar: 1.0 mm.

***Aedeagus***. (Fig. 6) Median lobe long and slender, moderately sclerotized, with a large, elongate membranous gonopore near apex; basal 1/5 doubly twisted; near apical 1/2 curved to one side in lateral view, in ventral view almost straight; tegmen located near apical 1/3 of median lobe (= penis), tegminal plate short and M-shaped in ventral view, tegminal strut large, long and flattened, extending nearly to base of median lobe.

**Figure 7.**
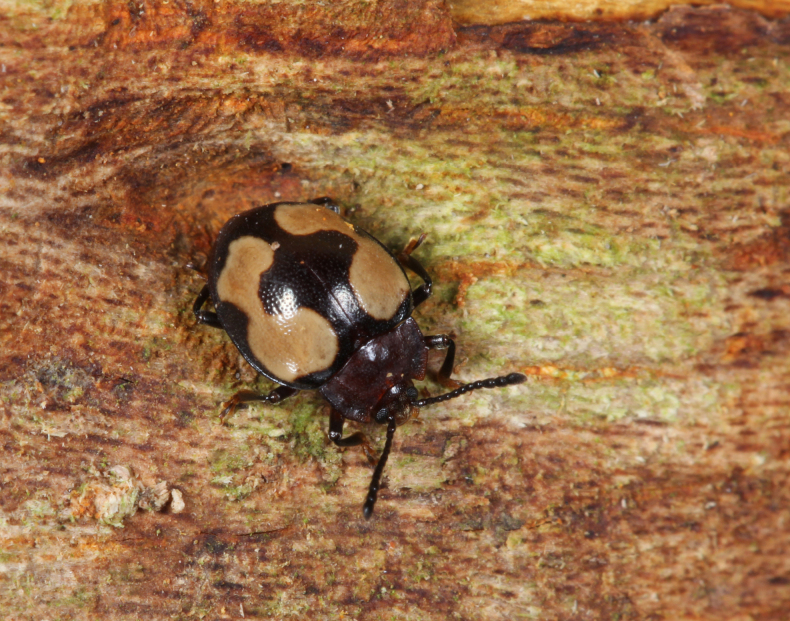
A living female of *M. speciosus* sp. nov.

**Female. *Habitus*** (Figs 3D–F, 7) similar to male in appearance. However, elytra sides gradually converging from apical 1/3 length of elytra to apex; ventrite 5 with posterior margin pointed and curved at apex; the non-punctate areas on ventrites 1–3 are reduced and raised compared to those in male.

***Measurements* (in mm)**. BL 4.1–4.5, BH 2.2–2.4, PL 1.1–1.3, PW 1.6–2.1, EL 3.3–4.1, EW 2.8–3.1, BH/BL 0.5, PL/PW 0.6–0.7, EL/EW 1.2–1.3, EL/PL 3.0–3.2, EW/PW 1.5–1.8.

#### Etymology.

The specific epithet is the Latin adjective *speciosus*, meaning beautiful or showy, in reference to its brightly colored elytral maculae.

#### Distribution.

China: Fujian.

### Meilichius tomaszewskae

Taxon classificationAnimaliaColeopteraEndomychidae

Chang & Bi sp. nov.

B3521D99-6D06-53C6-8F8D-F57D0E531171

https://zoobank.org/BAC1BB76-11A2-4F5D-897D-B387A5D5D0FC

Figs 8, 9, 10, 11

#### Type material.

***Holotype***: China • ♂; **Yunnan**, Longchuan County (陇川县), Husa Township (户撒乡); 1770–1700 m; 12.VI.2017, Wen-Xuan Bi leg.; (CBWX); ***Paratypes***: China • 6♂♂; same collecting data as holotype; (CBWX).

#### Diagnosis.

While *Meilichius tomaszewskae* sp. nov. resembles *M. ampliatus*, *M. apicicornis*, *M. biplagiatus*, *M. brevicollis*, *M. callosus*, *M. fuscipes*, *M. nigricollis*, *M. pachycerus*, and *M. politus* in general appearance, it can be distinguished from all congeners by the presence of short pubescence on the elytra.

#### Description.

**Figure 8.**
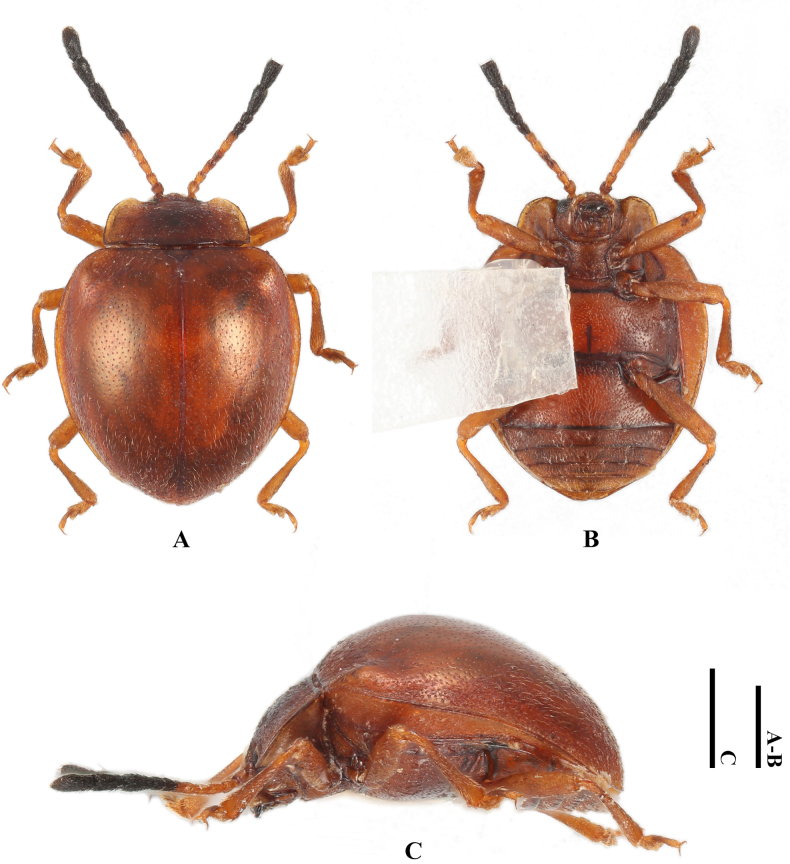
Habitus of *Meilichius tomaszewskae* sp. nov. (male). **A**. Dorsal view; **B**. Ventral view; **C**. Lateral view. Scale bars: 1.0 mm.

**Male. *Body*** (Fig. 8) broadly oval, convex, rather shiny, orange-brown with antennomeres 7–11 and eyes black; surfaces covered with short pubescence.

***Head***. Antenna composed of 11 antennomeres, with scape rather short and stout, 2.0× longer than pedicel; pedicel longer than wide; antennomere 3 ~ 1.5× longer than antennomere 2 and slightly longer than antennomere 4; antennomeres 4–6 longer than wide and gradually shorter; club composed of three antennomeres, narrow and loosely articulated; antennomere 9 as long as 7 and 8 combined and slightly longer than antennomere 10; terminal antennomeres 3.0× as long as wide. Maxilla with terminal palpomere 2.0× as long as wide, sides tapering toward apex, obliquely truncate apically.

***Thorax***. Pronotum with anterior and lateral margins very narrowly bordered; disc weakly convex, median furrow absent. Pronotal surface polished between punctures, punctation rather dense and fine; lateral sulci distinct, linear, extending to 1/5 of pronotal length; basal sulcus faint and nearly invisible. Prosternal process widely separates front coxae, ~ 2.0× as wide as longest coxal diameter and narrower than intercoxal process of mesoventrite, narrowest near mid-length, strongly widening behind front coxae toward apex, weakly rounded at apex. Elytral sides strongly curved, widest near mid-length, gradually converged apically; elytral epipleuron broad, abruptly tapered beyond mid-length and obsolete at apex. Humeri moderately prominent; elytral surface polished between punctures, punctation dense, coarser than pronotal ones.

**Figure 9.**
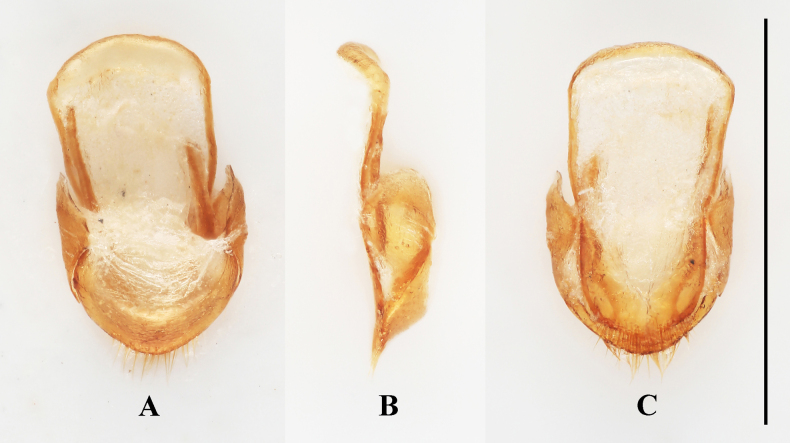
Male genital segment of *Meilichius tomaszewskae* sp. nov. **A**. Dorsal view; **B**. Lateral view; **C**. Ventral view. Scale bar: 1.0 mm.

***Abdomen***. Ventrite 1 slightly shorter than three subsequent ventrites combined; ventrites 2–4 gradually shorter in length. Ventrite 5 with posterior margin subtruncate at apex. Male genital segment (Fig. 9) with paired apophyses, fused apically, forming a U-shape, and with an additional pair of struts laterally of different length, one being long and the other short; subgenital plate wide and subrectangular; sternite IX narrow, truncate and setose at apex; tergite IX comprises two large laterotergites connected by a wide intervening membrane; tergite X narrowly rounded apically.

**Figure 10.**
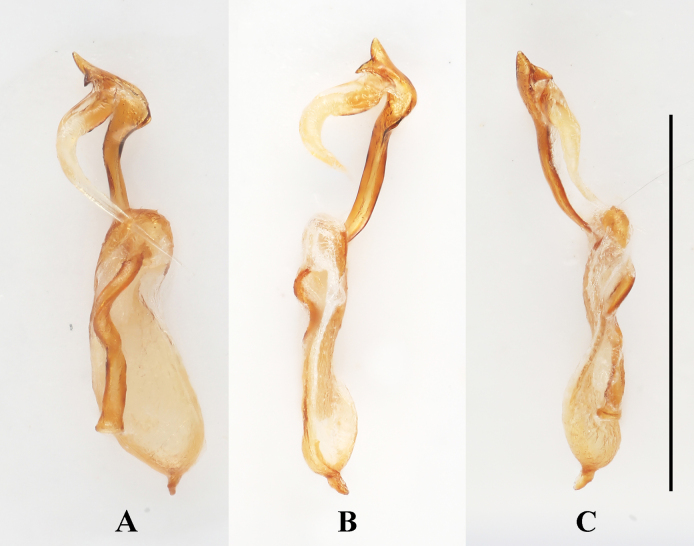
Aedeagus of *Meilichius tomaszewskae* sp. nov. **A**. Ventral view; **B**. Left lateral view; **C**. Right lateral view. Scale bar: 1.0 mm.

***Aedeagus*** (Fig. 10). Median lobe long and slender, sclerotized; ramified apically, with a large, elongate membranous gonopore near apex; basal 1/2 strongly twisted; apical 1/2 flattened and curved to one side in lateral view, in ventral view almost straight, abruptly widened at apical 1/5 into a C-shape; Tegmen located near 1/2 length of median lobe, tegminal plate short and arcuate in ventral view, tegminal strut large, long and flattened, extending beyond base of median lobe.

**Female**. Unknown.

***Measurements* (in mm)**. BL 3.4–3.8, BH 2.2–2.3, PL 0.8–1.2, PW 1.5–2.0, EL 3.0–3.5, EW 2.2–3.0, BH/BL 0.6, PL/PW 0.5–0.6, EL/EW 1.2–1.4, EL/PL 2.9–3.8 EW/PW 1.5.

#### Etymology.

The specific name pays tribute to Wioletta Tomaszewska, a Polish coleopterist, in acknowledgment of her work and significant contributions to the study of Endomychidae taxonomy.

**Figure 11.**
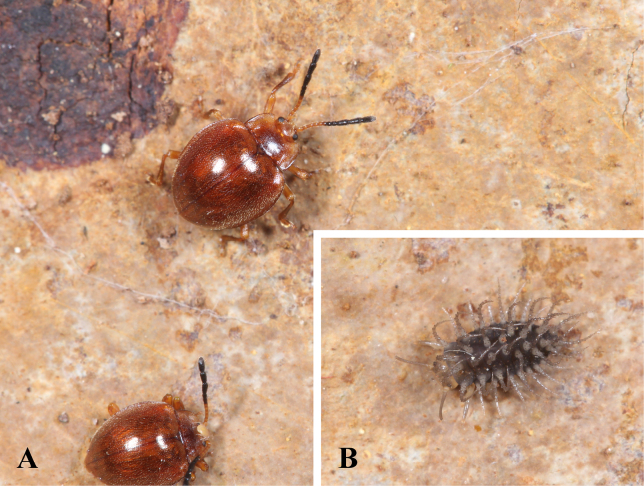
Living male of *M. tomaszewskae* sp. nov. **A**. Adult; **B**. Larva.

#### Distribution.

China: Yunnan.

##### Review of the species of *Meilichius* Gerstaecker

### Meilichius aeneoniger

Taxon classificationAnimaliaColeopteraEndomychidae

Strohecker, 1944

6CD006BA-46AE-5047-95F9-1E75935D7528

Fig. 12

Meilichius aeneoniger Strohecker, 1944: 141.

#### Type material examined.

***Paratype***: India • 1 sex undetermined; glued on a plastic sheet with six labels as follows: “Para-type” “Co-type” “Nilgiri Hills” “1946.178” “PARATYPE *Meilichius aeneoniger* Strohecker” “NHMUK 016510425”; (NHMUK).

#### Diagnosis.

Body approximately circular, strongly convex, surfaces smooth, shiny with metallic luster. Dorsal surface mostly copper-green, antenna, pronotal sides, elytral humeri, and legs brown. Elytra widest near mid-length; humeri barely prominent; elytral punctation distinctly dense and fine.

**Figure 12.**
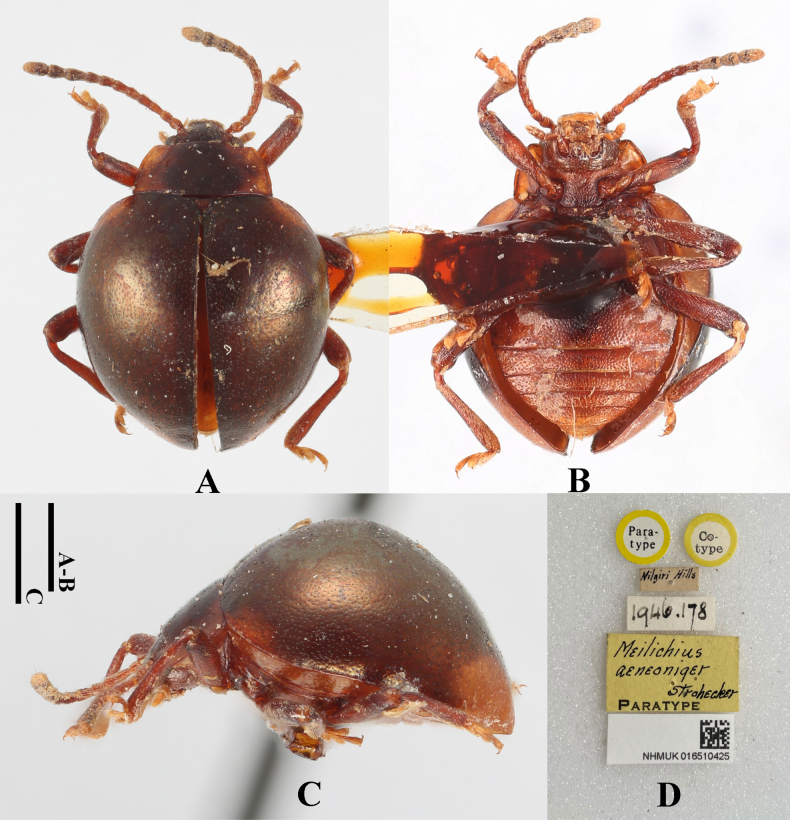
Paratype specimens of *Meilichius aeneoniger*. **A**. Dorsal view; **B**. Ventral view; **C**. Lateral view; **D**. Labels. Scale bars: 1.0 mm.

***Measurements* (in mm)**. BL 3.7, EW 2.9.

#### Distribution.

Southern India.

### Meilichius ampliatus

Taxon classificationAnimaliaColeopteraEndomychidae

(Gorham, 1875)

6D8ACE6A-E9E1-5CA0-813C-7205B2D3C924

Fig. 13

Thelgetrum ampliatum Gorham, 1875: 314.

#### Type material examined.

***Lectotype*** (here designated): Philippines • 1♂; glued on a card with eight labels as follows: “SYN-TYPE” “Type” “Gorham Type” “Phillipine Isles” “F. Chapuis” “Gorham Coll. 91–50” “T. ampliatum Gorham” “NHMUK 016510424”; (NHMUK).

#### Additional material examined.

Philippines • 2 sex undetermined; Surigao Mindanao Baker; Philippine Is. C. F. Baker. 1920–79; (NHMUK).

#### Diagnosis.

Body broadly oval, moderately convex, surfaces smooth, shiny. Dorsal surface mostly reddish brown, antennomeres 1–2 and 11 pale yellow; 3–10 brown to brownish-black. Pronotal lateral margins subparallel, from apical third strongly converging toward the apex. Elytra widest behind the mid-length; humeri prominent; elytral punctuation dense and fine.

**Figure 13.**
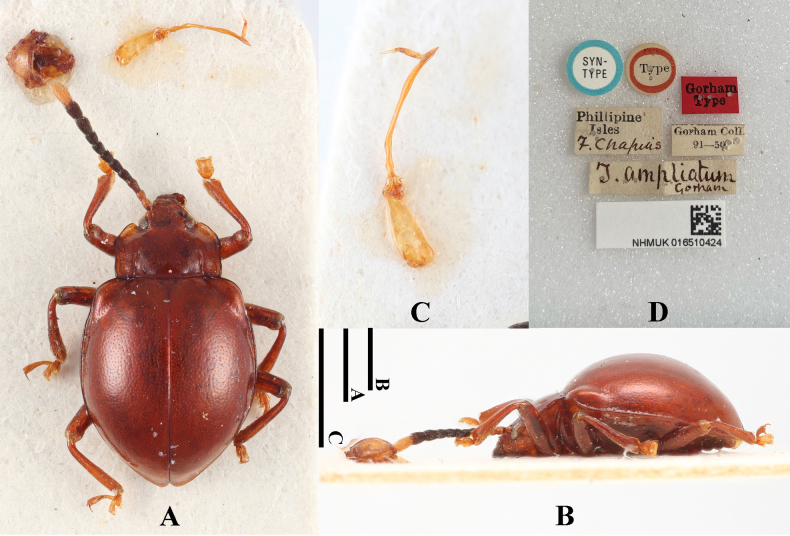
Lectotype of *Meilichius ampliatus* (here designated). **A**. Dorsal view; **B**. Lateral view; **C**. Aedeagus; **D**. Labels. Scale bars: 1.0 mm.

***Measurements* (in mm)**. BL 3.4–4.2, EW 2.4–2.7.

#### Distribution.

Philippines (Mindanao).

#### Remarks.

Gorham (1875) described *Thelgetrum ampliatum* based on a single specimen but did not explicitly designate it as the “holotype.” The first author examined this specimen housed in the Natural History Museum, London (NHMUK), and confirmed that its morphological characters and label data match the original description. Therefore, to clarify and stabilize its taxonomic status, the specimen bearing the historical “TYPE” label is hereby formally designated as the lectotype of *Meilichius ampliatus* (Gorham, 1875).

### Meilichius apicicornis

Taxon classificationAnimaliaColeopteraEndomychidae

Arrow, 1920

9828ABB5-DB7A-51C9-A2E8-695A74658422

Fig. 14

Meilichius apicicornis Arrow, 1920a: 72.

#### Type material examined.

***Lectotype*** (here designated): Borneo • 1♂; glued on a card with seven labels as follows: “SYN-TYPE” “Type H.T.” “Mt. Matang, W. Sarawak. G. E. Bryant. 16. XII. 13.” “D6 16” “G. Bryant Coll. 1919–147” “Meilichius apicicornis type arrow” “NHMUK 016510365”; (NHMUK); ***Paralectotypes*** (here designated): Borneo • 1♂; glued on a card with four labels as follows: “SYN-TYPE” “Mt. Matang, W. Sarawak. G. E. Bryant. XII. 1913” “G. Bryant Coll. 1919–147” “Meilichius apicicornis co-type arrow”; (NHMUK); Borneo • 1 sex undetermined; glued on a card with three labels as follows: “SYN-TYPE” “Mt. Matang, W. Sarawak. G. E. Bryant. 8. II. 14” “G. Bryant Coll. 1919–147”; (NHMUK) Borneo • 1 sex undetermined; ditto; (NHMUK); Borneo • 1 sex undetermined; ditto except “2. 1914”; (NHMUK).

#### Diagnosis.

Body short-ovate, strongly convex, surfaces smooth, shiny. Dorsal surface mostly reddish brown, antennomere 1–4 and terminal one ferruginous; 5–10 black. Elytra widest near mid-length; humeri moderately prominent; elytral punctuation distinctly dense and coarse.

**Figure 14.**
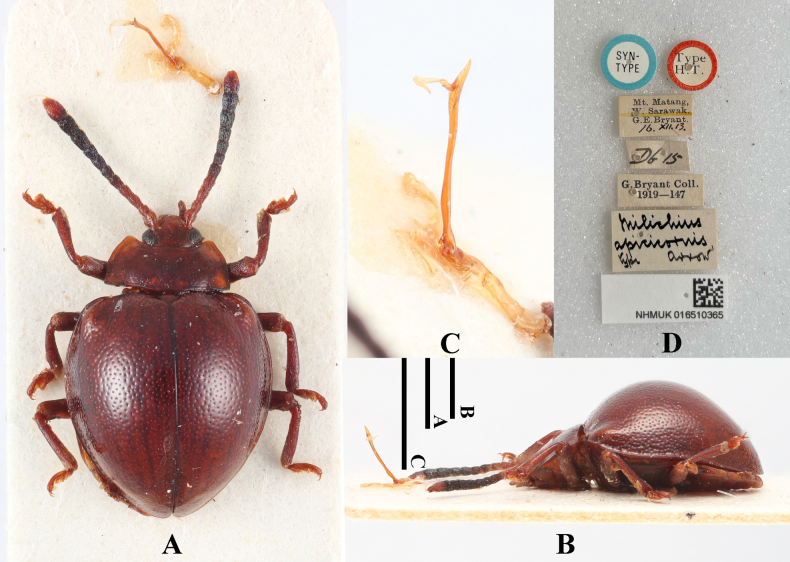
Lectotype of *Meilichius apicicornis* (here designated). **A**. Dorsal view; **B**. Lateral view; **C**. Aedeagus; **D**. Labels. Scale bars: 1.0 mm.

***Measurements* (in mm)**. BL 3.8–4.4, EW 2.6–3.1.

#### Distribution.

Malaysia (Sarawak).

#### Remarks.

The first author has examined all the type specimens of *M. apicicornis* deposited in the Natural History Museum in London. According to the original description by Arrow (1920a), “A series of specimens were taken by Mr. Bryant.” This indicates that the description of this new species was based on multiple specimens. Indeed, the first author examined five specimens of this species bearing syntype labels. Herein we designate the male specimen with collection number NHMUK 016510365 as the lectotype to stabilize the taxonomic status of this species.

### Meilichius biplagiatus

Taxon classificationAnimaliaColeopteraEndomychidae

Arrow, 1920

9303B090-23D1-596E-8394-95E32A08CFF0

Fig. 15

Meilichius biplagiatus Arrow, 1920a: 73.

#### Type material examined.

***Holotype***: Borneo • ♂; glued on a card with seven labels as follows: “Holotype” “Holo-type” “Type H. T.” “59564” “Borneo Pengaron” “Doherty” “Frv Coll. 1905.100.” “Meilichius biplagiatus type Arrow” “NHMUK 016510368”; (NHMUK).

#### Additional material examined.

Borneo • 1 sex undetermined; Sandakan; Brit. N. Borneo. C. F. Baker. 1919–2; Sandakan Borneo Baker; 9460; Meilichius biplagiatus. arr. Compared with type. G.J.A.; (NHMUK); Borneo • 1 sex undetermined; Sandakan Borneo Baker; Sandakan; Brit. N. Borneo. C. F. Baker. 1919–2; (NHMUK).

#### Diagnosis.

Body short-ovate, strongly convex, surfaces smooth, shiny. Dorsal surface reddish brown, antennomere 1–11 pale brown. Elytra widest near mid-length; humeri strongly prominent; elytral punctuation dense and rather fine.

**Figure 15.**
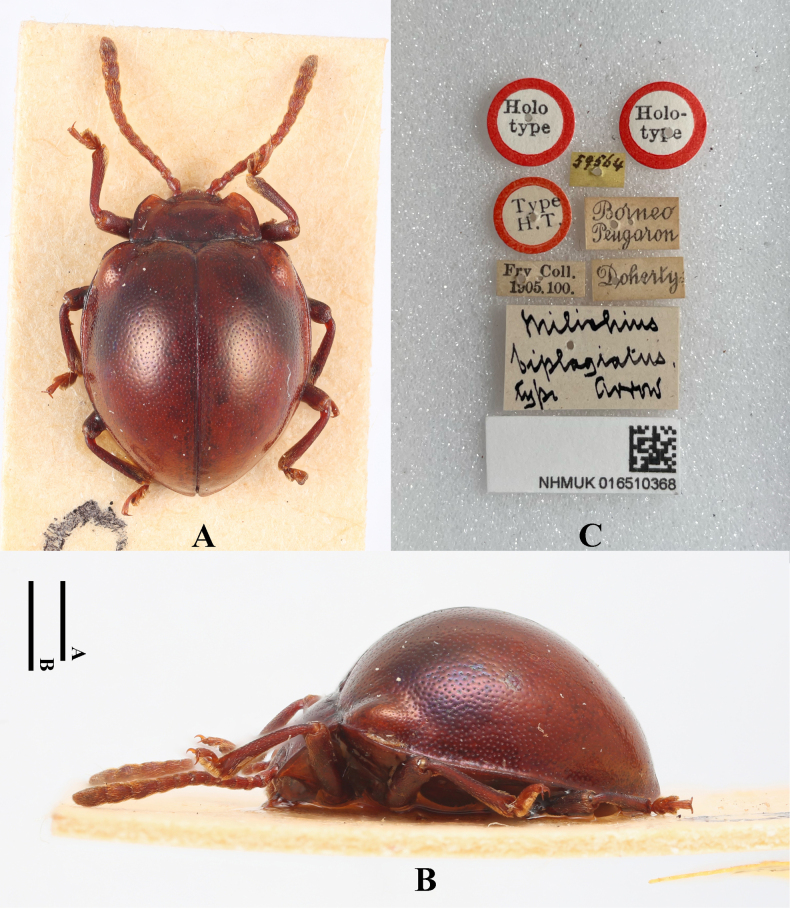
Type specimens of *Meilichius biplagiatus*. **A**. Dorsal view; **B**. Lateral view; **C**. Labels. Scale bars: 1.0 mm.

***Measurements* (in mm)**. BL 4.4–4.5, EW 3.3–3.4.

#### Distribution.

Indonesia (S. Borneo) and Malaysia (N. Borneo).

### Meilichius brevicollis

Taxon classificationAnimaliaColeopteraEndomychidae

Arrow, 1920

594B9FC1-7328-58DF-9FD7-9B1E261CAE36

Fig. 16

Meilichius brevicollis Arrow, 1920a: 72.

#### Type material examined.

***Holotype***: Borneo • ♀; glued on a card with seven labels as follows: “Holo-type” “Type H. T.” “Borneo Pengaron” “Doherty” “Frv Coll. 1905.100.” “Meilichius brevicollis type arrow” “NHMUK 016510367”; (NHMUK).

#### Additional material.

Borneo • 1 ♀; Sarawak: 1907–1909. C. J. Brooks. B. M. 1936-681; (NHMUK); Borneo • 1 ♀; Sarawak, Bidi. 1908-9. C. J. Brooks. B. M. 1929–551; (NHMUK).

#### Diagnosis.

Body short-ovate, strongly convex, surfaces smooth, shiny. Dorsal surface mostly reddish brown, antennomeres 1–4 brown, 5–10 and the basal half of the terminal one black. Elytra widest near mid-length; humeri weakly prominent; elytral punctuation dense and rather fine.

**Figure 16.**
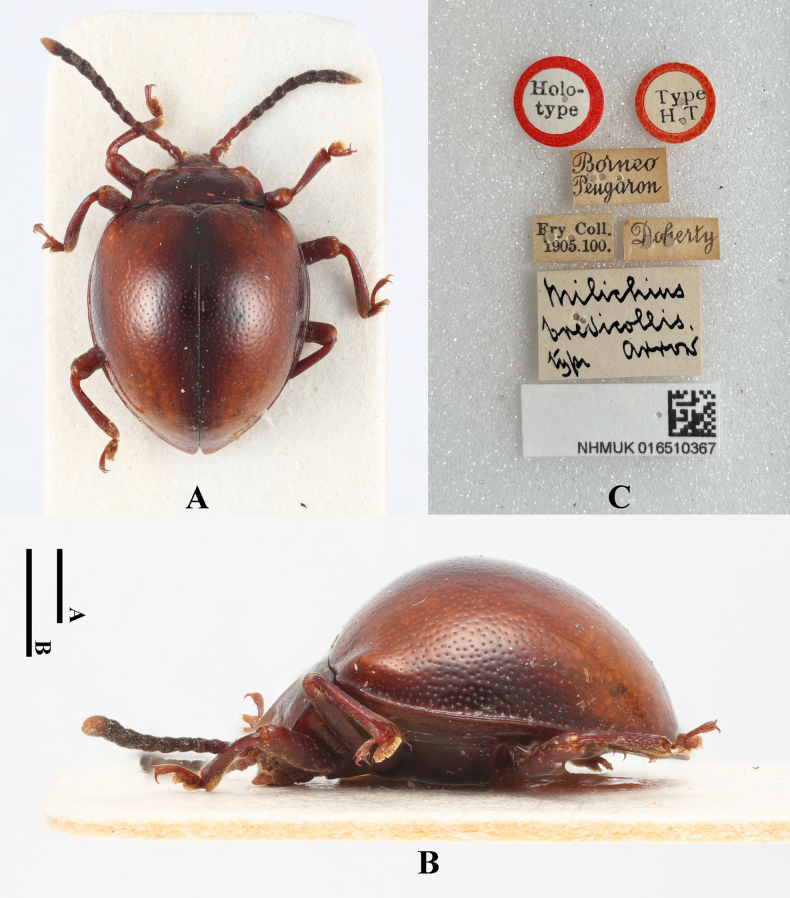
Type specimens of *Meilichius brevicollis*. **A**. Dorsal view; **B**. Lateral view; **C**. Labels. Scale bars: 1.0 mm.

***Measurements* (in mm)**. BL 4.1–4.6, EW 3.1–3.6.

#### Distribution.

Indonesia (S. Borneo) and Malaysia (Sarawak).

### Meilichius callosus

Taxon classificationAnimaliaColeopteraEndomychidae

Pic, 1930

0404B41D-7ACE-58B4-861D-5C922748DE1B

Fig. 17

Meilichius callosus Pic, 1930: 19.

#### Material examined.

Borneo • 1 sex undetermined; B. N. Borneo, nr. Kinabalu, Kabayau, 600. 13: 5: 1929; Meilichius callosus. Pic Determined from description. G. J. A.; NHMUK 016510423; (NHMUK); Borneo • 1 sex undetermined; B. N. Borneo, nr. Kinabalu, Kabayau, 600. 13: 5: 1929; Meilichius callosus. Pic Determined from description. G. J. A.; Ex F. M S. Museum B. M. 1955-354.; (NHMUK); Borneo • 1 sex undetermined; B. N. Borneo, nr. Kinabalu, Kabayau, 600. 13: 5: 1929; Ex F. M S. Museum B. M. 1955-354.; (NHMUK).

#### Diagnosis.

Body short-ovate, strongly convex, surfaces smooth, shiny. Dorsal surface pale brown, antennomeres 1–7 and the terminal one brown, antennomeres 8–10 black; humeral calli orange. Elytra widest near mid-length; humeri strongly prominent; elytral punctuation dense and coarse.

**Figure 17.**
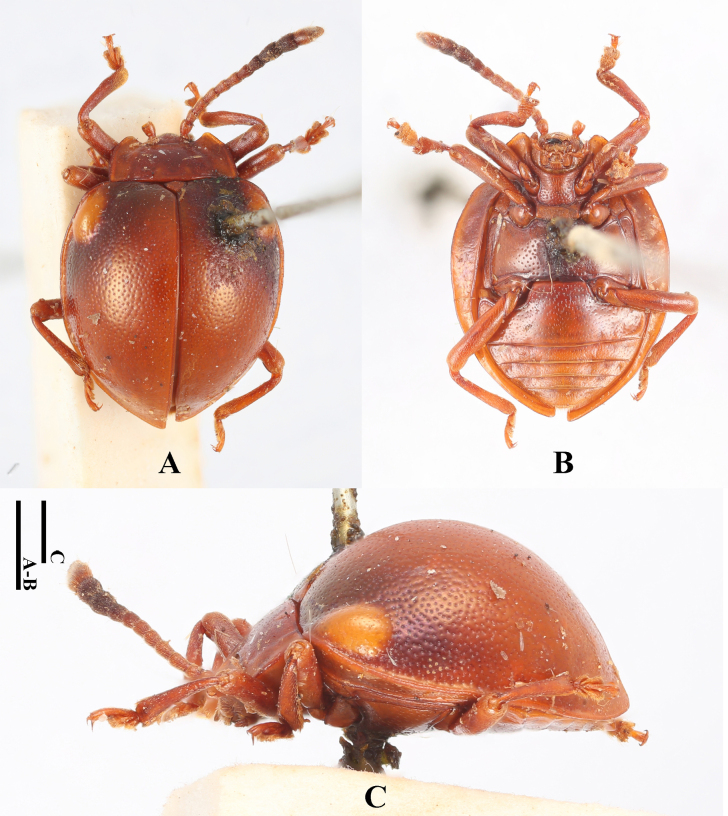
Habitus of *Meilichius callosus* (NHMUK 016510423). **A**. Dorsal view; **B**. Ventral view; **C**. Lateral view. Scale bars: 1.0 mm.

***Measurements* (in mm)**. BL 4.6–4.7, EW 3.7–3.8.

#### Distribution.

Malaysia (N. Borneo).

### Meilichius erotyloides

Taxon classificationAnimaliaColeopteraEndomychidae

Strohecker, 1951

BD6A95DC-16D7-5BCC-9E26-B6A39340451A

Fig. 18

Meilichius erotyloides Strohecker, 1951: 166.

#### Type material.

***Holotype***: China • sex undetermined; glued on a card with four labels as follows: “Ta Han, Hainan Id VI-24-35” “L. Gressitt Collection” “*Meilichius Erotyloides* Strohecker TYPE” “California Academy of Sciences Type No. 8363”; (CAS).

**Figure 18.**
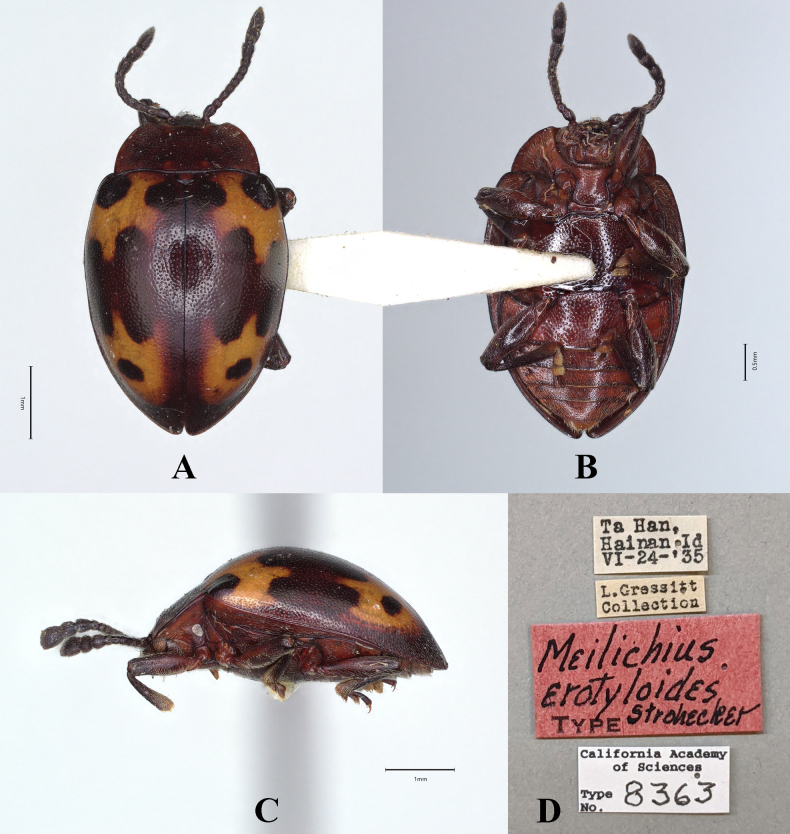
Type specimens of *Meilichius erotyloides*. **A**. Dorsal view; **B**. Ventral view; **C**. Lateral view; **D**. Labels. Scale bars: 0.5 mm (**B**), 1.0 mm (**A, C**). (Photographed by Rachel Diaz-Bastin, CAS).

#### Diagnosis.

Body elongate oval, moderately convex, surfaces smooth, shiny. Dorsal surface mostly black, pronotum deep red; elytral maculae yellow. Elytra widest near mid-length; humeri barely prominent; elytral punctuation dense and coarse. Each elytron with two irregular maculae; anterior elytral macula narrow, anterior margin enclosing humerus, posterior margin with three deep U-shaped concavities; posterior elytral macula large, anterior margin with three sharp teeth, posterior margin rounded, with a black spot at the center.

***Measurements* (in mm)**. BL 4.5.

#### Distribution.

China (Hainan).

#### Remark.

This species was examined in the present study with holotype photo observations, and the description and information presented here based on Strohecker (1951).

### Meilichius expetitus

Taxon classificationAnimaliaColeopteraEndomychidae

Gorham, 1885

364DB73D-D879-5ACE-9561-929F5AE26161

Meilichius expetitus Gorham, 1885: 523.

#### Type material.

“A single specimen taken in August 1878 by Doct. O. Beccari.”

#### Diagnosis.

Body short-ovate strongly convex, surface smooth, shiny. Antennomeres 1–4 ferruginous, 5–11 black; pronotum and elytral humeri ferruginous; elytra bright violet. Elytra widest near mid-length; humeri weakly prominent; elytral punctuation sparse and coarse.

***Measurements* (in mm)**. BL 4.5.

#### Distribution.

Sumatra.

#### Remark.

This species was not examined in the present study, nor were the type specimen photographs available. The description and information presented here are based on Gorham (1885).

### Meilichius fasciatus

Taxon classificationAnimaliaColeopteraEndomychidae

(Heller, 1898)

362AC381-28D9-54D4-9302-D9A82D4D93F2

Milichius fasciatus Heller, 1898: 40.

#### Type material.

“Southern Celebes, Lompo Batang, X, 1895, Drs. Sarasin; same locality, altitude 3000 ft., III 1896, H. Fruhstorfer leg. Mus. Dresden No. 10913 & Mus. Tring.”

#### Diagnosis.

Body approximately circular, strongly convex, surfaces smooth, shiny with strong metallic luster. Antennae, head, pronotum, and legs brown to black, elytra blue-black, elytral maculae orange. Elytra widest near mid-length; humeri barely prominent; elytral punctuation distinctly dense and rather coarse. Each elytron with one finger-like transverse macula (based on Heller 1898).

***Measurements* (in mm)**. BL 4.5–5.5.

#### Distribution.

Indonesia (Sulawesi).

#### Remark.

This species was not examined in the present study, nor were type specimen photographs available. The description and information presented here are based on Heller (1898).

### Meilichius ferrugineus

Taxon classificationAnimaliaColeopteraEndomychidae

Frivaldszky, 1883

DAAC8975-9C9A-5464-8DE8-571DD72448F5

Fig. 19

Meilichius ferrugineus Frivaldszky, 1883: 132.

#### Material examined.

Borneo • 1 sex undetermined; D6 16; Mt. Matang, W. Sarawak. G. E. Bryant. 24. 1. 14.; G. Bryant Coll. 1919–147; Determined from description. G. J. A. Meilichius ferrugineus Frival.; NHMUK 016510366; (NHMUK); ditto except “23. 1. 14.” (NHMUK); Borneo • 1 sex undetermined; Sandakan, N. Borneo. W. B. Pryer. B. M. 1925–264.; (NHMUK); Borneo • 1 sex undetermined; Sandakan, N. Borneo.; Sandakan, N. Borneo. W. B. Pryer. B. M. 1925–264.; (NHMUK); Borneo • 1 sex undetermined; Sandakan, N. Borneo. W. B. Pryer. B. M. 1925–264.; ? Cymbachus sp. n. notiu Gorh. coll.; (NHMUK); Borneo • 1 sex undetermined; Sandakan, N. Borneo.; Sandakan, N. Borneo. W. B. Pryer. B. M. 1925–264.; Meilichius ferrugineus Frival. G. J. Arrow det.; (NHMUK); Borneo • 1 sex undetermined; Sarawak: 4^th^ Division Gn. Mulu NP.; P. M. Hammond & J. E. Marshall v-viii. 1978 B. M. 1978–49; nr. Base Camp 50–100 m.; Meilichius sp. P. M. Hammond det. 198? (invisible handwriting); Meilichius ferrugineus (Frivaldszky) Det. W. Tomaszewska 2004; (NHMUK); Borneo • 1 sex undetermined; P. M. Hammond & J. E. Marshall v-viii. 1978 B. M. 1978–49; Sarawak: 4^th^ Division Gn. Mulu NP.; Meilichius ferrugineus (Frivaldszky) Det. W. Tomaszewska 2004; (NHMUK).

**Figure 19.**
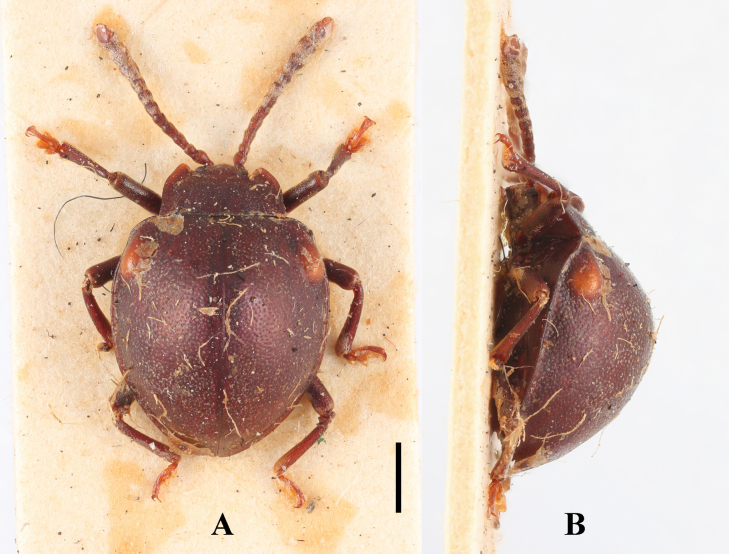
Habitus of *Meilichius ferrugineus*. **A**. Dorsal view; **B**. Lateral view. Scale bar: 1.0 mm.

#### Diagnosis.

Body short-ovate strongly convex, surfaces smooth, shiny with metallic luster. Antennae almost brown, pronotum and elytra violet-blue, legs (except tarsi) brown, elytral humeri and tarsi orange. Elytra widest near mid-length; humeri moderately prominent; elytral punctuation distinctly dense and coarse.

***Measurements* (in mm)**. BL 4.6–5.0, EW 3.2–3.5.

#### Distribution.

Malaysia (Sabah).

### Meilichius fuscipes

Taxon classificationAnimaliaColeopteraEndomychidae

Arrow, 1920

9B847A30-1931-5EDC-8F6E-1A3ACCB09127

Fig. 20

Meilichius fuscipes Arrow, 1920a: 70.

#### Type material examined.

***Holotype***: Sumatra • ♂; glued on a card with five labels as follows: “Holo-type” “Type H. T.” “Padang. Sidempoean. Sumatra. Ericson. 99–94.” “Meilichius fuscipes. Type arrow” “NHMUK 016510363”; (NHMUK).

#### Diagnosis.

Body short-ovate, strongly convex, surfaces smooth, shiny. Antennomeres 1–10 deep brown to black, terminal one pale yellow; elytra bright ferrugineus red, pronotum slightly darker, legs dark brown. Elytra widest near behind mid-length; humeri distinctly prominent; elytral punctuation sparse and fine.

**Figure 20.**
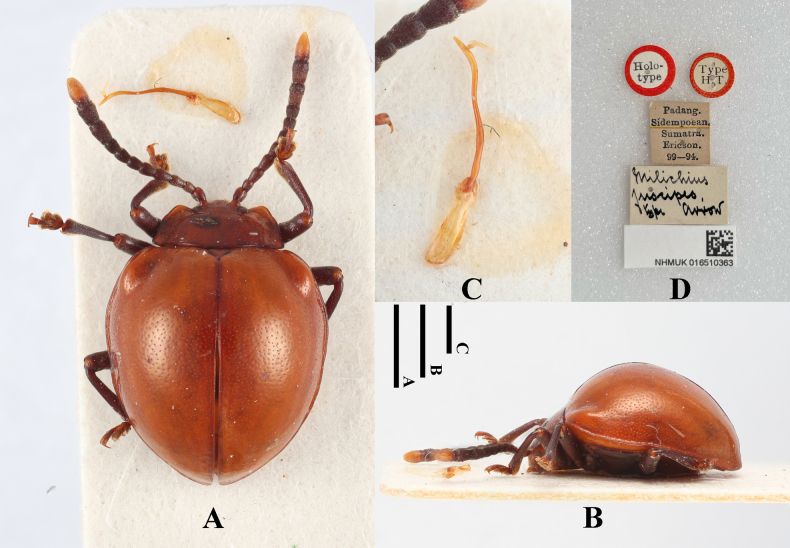
Holotype specimens of *Meilichius fuscipes*. **A**. Dorsal view; **B**. Lateral view; **C**. Aedeagus; **D**. Labels. Scale bars: 1.0 mm.

***Measurements* (in mm)**. BL 4.6, EW 3.2.

#### Distribution.

Sumatra (Padang).

### Meilichius geminatus

Taxon classificationAnimaliaColeopteraEndomychidae

Strohecker, 1958

E40867A0-D76D-5AFA-BE5C-91FC939B062C

Meilichius geminatus Strohecker, 1958: 45

#### Type material.

“Holotype.—A specimen of undetermined sex from Luzon. In the collection of the Zoologisches Museum der Humboldt-Universitat, Berlin.” [not examined]

#### Diagnosis.

Body approximately circular, strongly convex, surfaces smooth, shiny. Dorsal surface mostly ferruginous, antennomeres 1–4 pale brown, 5–11 black; elytral maculae and legs black. Elytra widest near behind mid-length; humeri weakly prominent; elytral punctuation dense and coarse. Each elytron with two pairs of oval spots, the anterior and posterior pairs widely separates.

***Measurements* (in mm)**. BL 3.5.

#### Distribution.

Philippines (Luzon).

#### Remark.

This species was not examined in the present. The description and information presented here are based on Strohecker (1958).

### Meilichius impressicollis

Taxon classificationAnimaliaColeopteraEndomychidae

Strohecker, 1943

6BB7AA17-FE87-5AA4-AAA8-31974BD652B6

Fig. 21

Meilichius impressicollis Strohecker, 1943: 389.

#### Type material.

***Holotype***: Philippines • sex undetermined; glued on a card with four labels as follows: “Mt. Makiling Luzon. Baker”“Type” “TypeNo 55889 U S N M” “*Meilichius impressicollis* Strohecker”; (USNM).

**Figure 21.**
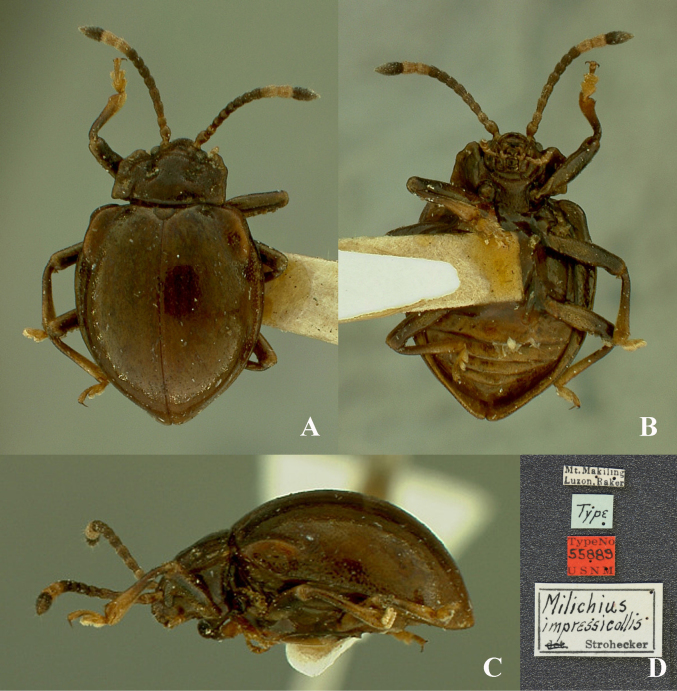
Habitus of *Meilichius impressicollis*. **A**. Dorsal view; **B**. Ventral view; **C**. Lateral view; **D**. Labels. Image credit: University of Georgia Herbarium (GA), specimen record UGA5071094 (iDigBio, accessed 4 Dec 2025). Image licensed under CC0.

#### Diagnosis.

Body broadly oval, moderately convex, surfaces smooth, shiny with aeneous luster. Dorsal surface mostly ferruginous; antennomeres 6–8 and 11 black; 9 and 10 pale yellow; elytral humeri pale ferruginous. Elytra widest near mid-length; humeri distinctly prominent, subcarinate laterally; elytral punctuation sparse and fine.

#### Distribution.

Philippines (Luzon).

#### Remark.

This species was examined in the present study with holotype photo observations, and the description and information presented here are based on Strohecker (1943).

### Meilichius javanicus

Taxon classificationAnimaliaColeopteraEndomychidae

Csiki, 1900

EF2B90D5-B6A5-55D5-81AD-7B185592E991

Fig. 22

Meilichius javanicus Csiki, 1900: 376.

#### Material examined.

Java • 1♂; Bowring. 63·47*; determined from description. G. J. A. Meilichius javanicus Csiki; NHMUK 016510426; (NHMUK).

**Figure 22.**
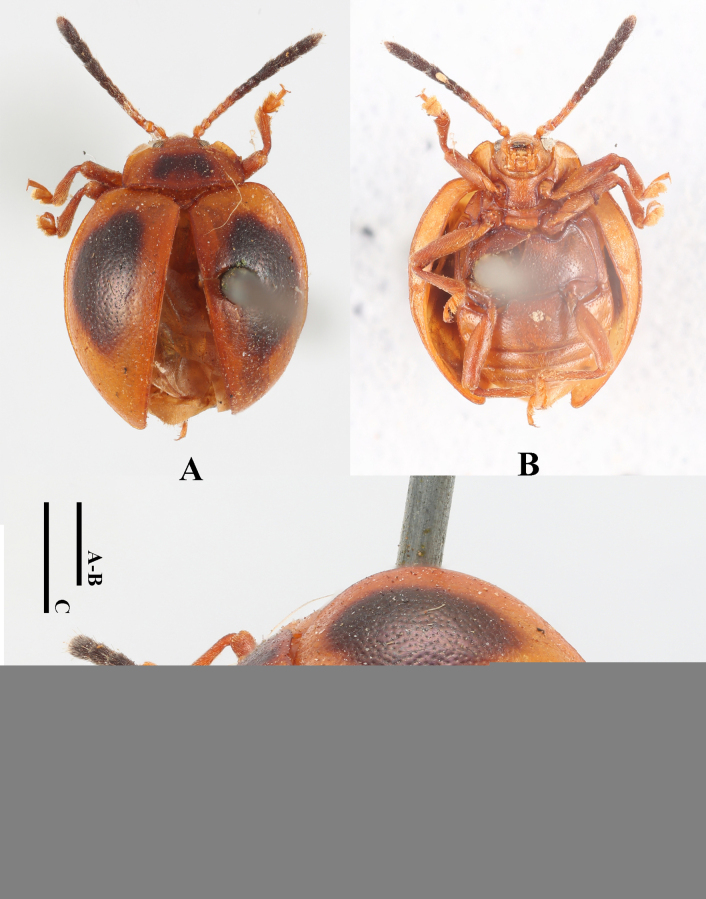
Habitus of *Meilichius javanicus*. **A**. Dorsal view; **B**. Ventral view; **C**. Lateral view. Scale bars: 1.0 mm.

#### Diagnosis.

Body approximately circular, strongly convex, surfaces smooth, shiny. Dorsal surfaces mostly pale ferruginous; antennomeres 6–11 dark brown, pronotal disc, elytral maculae black. Elytra widest near mid-length; humeri barely prominent; elytral punctuation distinctly dense and rather coarse. Each elytron with a single longitudinal half-oval shaped macula; the maculae broadly separated when elytra closed.

***Measurements* (in mm)**. BL 3.7, EW 3.2.

#### Distribution.

Java.

### Meilichius klapperichi

Taxon classificationAnimaliaColeopteraEndomychidae

Mader, 1941

895DDB05-D5B3-5A5B-A18B-A1D5DD50331D

Fig. 23

Milichius klapperichi Mader, 1941: 938.

#### Type material.

***Holotype***: China • sex undetermined; glued on a card with five labels as follows: “Kuatun (2300 m) 27, 40n. Br. 117, 40ö.L. J. Klapperich 7. 5. 1938 (Fukien)” “Milichius klapperichi det. Mader”“Holotypus” “Automontage photographed” “ZFMK-COL-1000132”; (ZFMK). ***Paratype***: China • sex undetermined; in Mader’s collection (Mader 1941).

**Figure 23.**
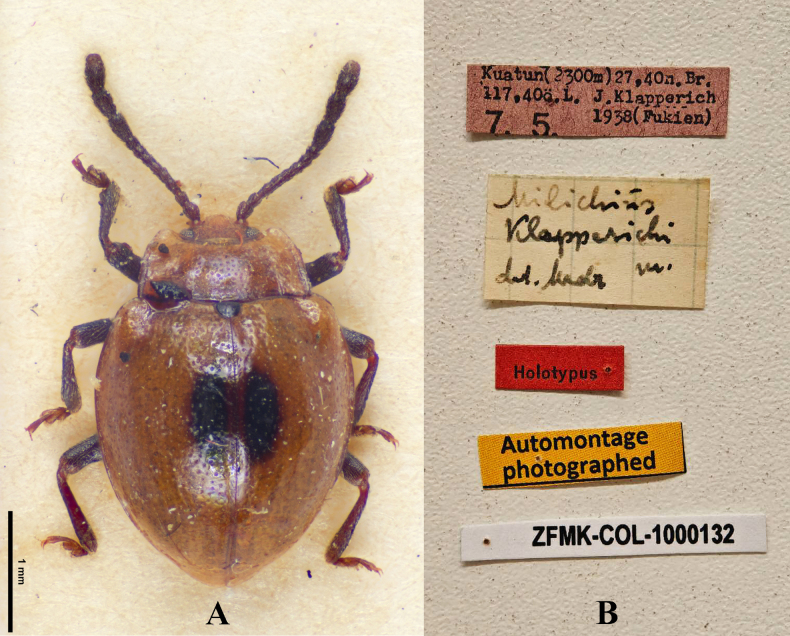
Holotype specimens of *Meilichius klapperichi*. **A**. Dorsal view; **B**. Labels. Scale bar: 1.0 mm. (Photographed by Dirk Ahrens, ZFMK).

#### Diagnosis.

Body broadly oval, strongly convex, surfaces smooth, shiny. Dorsal surface mostly pale ferruginous; antennae and legs dark reddish-brown to black, elytral maculae black. Elytra widest near mid-length; humeri weakly prominent; elytral punctuation dense and rather coarse. Each elytron with a single small longitudinal half-oval shaped macula; the maculae forming a single circular spot medially when elytra closed.

***Measurements* (in mm)**. BL 3.0–3.5.

#### Distribution.

China (Fujian).

#### Remark.

This species was examined in the present study with holotype photo observations, and the description and information presented here are based on Mader (1941).

### Meilichius multimaculatus

Taxon classificationAnimaliaColeopteraEndomychidae

Sasaji, 1970

C0ABC2BC-DAAB-57E9-9288-C9E270AB00FB

Meilichius multimaculatus Sasaji, 1970: 15.

#### Type material.

“Holotype. ♀, Penpuchi, Nantou Hsien, Formosa, 13. Vii. 1966, H. Kamiya leg.” [not examines]

#### Diagnosis.

Body elongate oval, moderately convex, surfaces smooth, shiny with coppery luster. Antennae black; pronotum deep reddish-brown, with lateral area rather broadly reddish; elytra yellowish brown with black complicated maculae; legs reddish brown. Elytra widest near mid-length; humeri prominent; elytral punctuation sparse and fine. Elytral maculae as follows: A large scutellar black patch narrows posteriorly and connects to a very narrow sutural black stripe. This stripe then broadens again to form a large preapical black area, which extends broadly forward along the outer margin, with its anterior margin dentate. The apical margins of the elytra are very narrowly reddish. The humeral black macula is transverse, with an excavated posterior border, and is separated from the scutellar macula by a narrow pale stripe. The lateral black macula is large, entirely touching the outer margin, and situated before the middle of the elytral length. On each elytron, two small black spots are transversely aligned at the same level. A pair of triangular, medium-sized maculae is located one-third from the apex and near the suture.

***Measurements* (in mm)**. BW 3.4, BH 1.5.

#### Distribution.

China (Taiwan).

#### Remark.

This species was not examined in the present. The description and information presented here are based on Sasaji (1970).

### Meilichius nigricollis

Taxon classificationAnimaliaColeopteraEndomychidae

Gerstaecker, 1857

C9DDAE63-BA08-5219-8293-6C2B27B0A55F

Figs 24, 25

Meilichius nigricollis Gerstaecker, 1857: 241.

#### Type material examined.

***Holotype***: Borneo • ♀; glued on a card with six labels as follows: “Pulo Pe. nang. Nigricollis gerst.” “*Meilichius nigricollis* Gerst. ♀” “TYPE” “Mus. Westerm.” “dissected & re-mounted May 1961 H. F. Strohecker” “zmuc00020050”; (NHMD).

**Figure 24.**
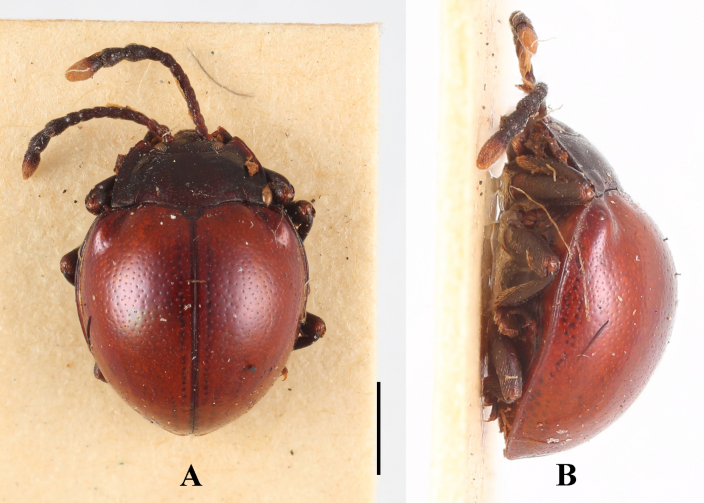
Habitus of *Meilichius nigricollis* (NHMUK 016510361). **A**. Dorsal view; **B**. Lateral view. Scale bar: 1.0 mm.

**Figure 25.**
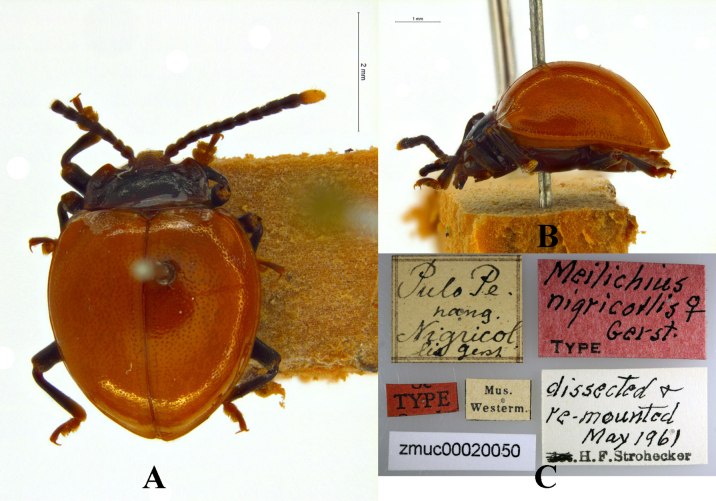
Holotype of *Meilichius nigricollis*. **A**. Dorsal view; **B**. Lateral view; **C**. Labels. Scale bars: 1.0 (**B**), 2.0 mm (**A**). Image credit: Natural History Museum of Denmark, Entomological Collection, specimen NHMD52459 (Scharff et al. 2025). Data accessed via GBIF.org, 4 Dec 2025. Image licensed under CC BY 4.0.

#### Additional material examined.

Borneo • 1 sex undetermined; Quop, W. Sarawak. II-III. 1914. G. E. Bryant.; G. Bryant Coll. 1919–147; NHMUK 016510361; (NHMUK); Borneo • 1 sex undetermined; Mt. Matang, W. Sarawak. G. E. Bryant. 31. 1. 14; G. Bryant Coll. 1919–147; determined from description. G. J. A. ? Meilichius nigricollis Gerst.; NHMUK 016510360; (NHMUK); Borneo • 1 sex undetermined; Quop, W. Sarawak. W. Sarawak. 2. III. 14.; D625; G. Bryant Coll. 1919–147; Determined from description. G. J. A. ? Meilichius nigricollis Gerst.; (NHMUK); Borneo • 1 sex undetermined; N. Borneo. Bettotan, Nr. Sandakan. Aug. 10^th^. 1927.; Ex F. M. 30 S. Museum. B. M. 1955-354.; (NHMUK); Borneo • 1 sex undetermined; N. BORNEO. Bettotan, Nr. Sandakan. Aug. 9^th^. 1927.; M. biplagiatus. arr. Compared with type. G. J. A.; Ex F. M. S. Museum. B. M. 1955-354.; (NHMUK); Malaysia • 1 sex undetermined; MALAYA SELANGOR, F. M. S. ul? kanocn (invisible handwriting) 30. 8. 1934; Ex F. M. S. Museum. B. M. 1955-354.; ? Meilichius nigricollis Gerst. R. J. W. Aldridge det. 1979.; (NHMUK).

#### Diagnosis.

Body short-ovate, strongly convex, surfaces smooth, shiny. Dorsal surface mostly ferruginous; antennomeres 1–10 dark reddish-brown to black, the apical half of the terminal one bright yellow; legs black. Elytra widest near mid-length; humeri distinctly prominent; elytral punctuation sparse and fine.

***Measurements* (in mm)**. BL 3.3–5.6, EW 2.3–4.3.

#### Distribution.

Malaysia and Indonesia.

#### Remarks.

Among the specimens of this species examined by the first author except for the holotype, only one specimen bears a label stating “Borneo” (NHMUK 016510361) and the single specimen bears a label stating: “Malaysia” have a black pronotum, a characteristic consistent with the original description and the holotype specimen, while the other specimens have reddish-brown pronotums. Among the latter, two bear identification labels annotated with a question mark by the identifier, suggesting that the identifier doubted the correct taxonomic placement of these specimens. It is also noteworthy that one specimen from Malaysia has a black pronotum, but its identification label is similarly marked with a question mark by the identifier.

### Meilichius ornatus

Taxon classificationAnimaliaColeopteraEndomychidae

Arrow, 1920

21309C8A-18A5-5EEE-A7F8-18935EFFD5E7

Figs 26, 27

Meilichius ornatus Arrow, 1920b: 332.

#### Type material examined.

***Lectotype*** (here designated): Laos • ♂; glued on a card with six labels as follows: “SYN-TYPE” “allo-Type” “1918–1” “Laos. Ban Na Lane. 2.I.1918. R.V. de Salvaza.” “Meilichius ornatus co-type arrow” “NHMUK 016412518”; (NHMUK). ***Paralectotype*** (here designated): Laos • 1♀; glued on a card with six labels as follows: “SYN-TYPE” “Type H. T” “1918–1” “Laos. Ban Na Lane. 2.I.1918. R.V. de Salvaza.” “Meilichius ornatus type arrow” “NHMUK 016412515”; (NHMUK).

**Figure 26.**
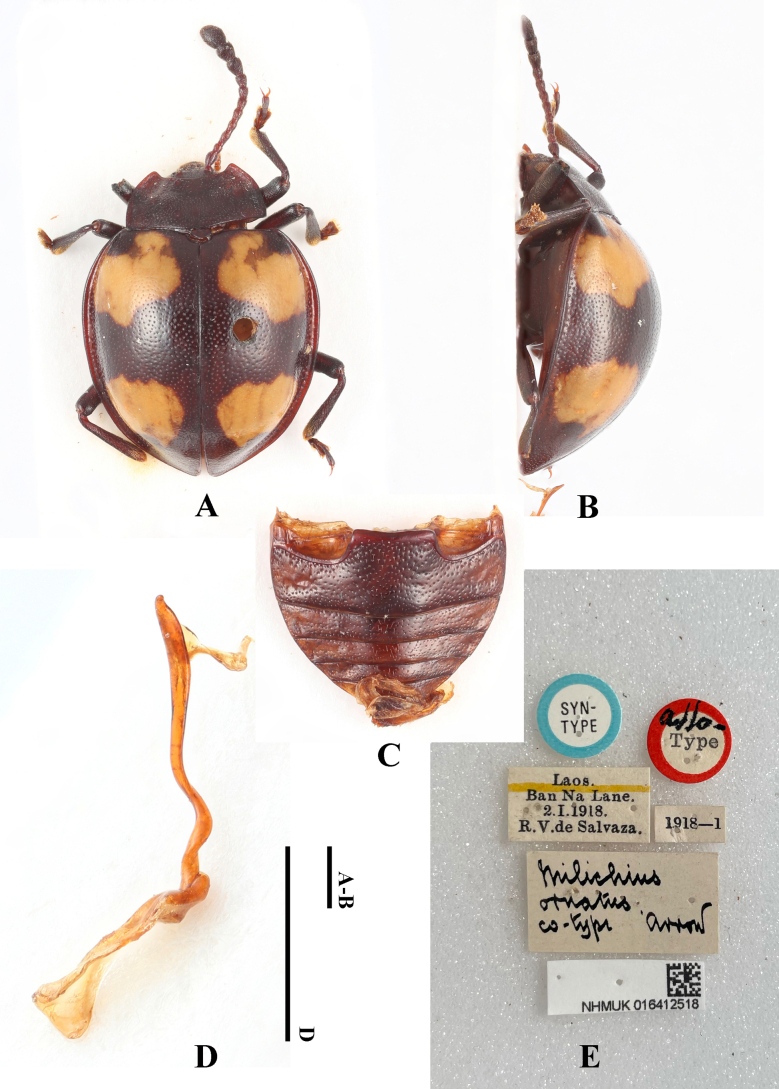
Lectotype of *Meilichius ornatus*, male. **A**. Dorsal view; **B**. Lateral view; **C**. Abdomen; **D**. Aedeagus; **E**. Labels. Scale bars: 1.0 mm.

**Figure 27.**
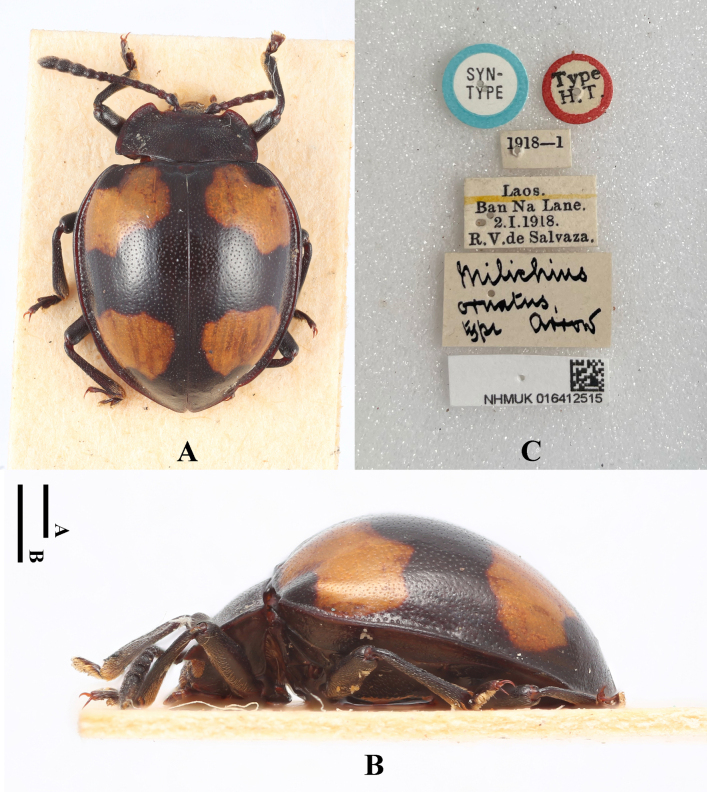
Paralectotype of *Meilichius ornatus*, female. **A**. Dorsal view; **B**. Lateral view; **C**. Labels. Scale bars: 1.0 mm.

#### Diagnosis.

Body short-ovate, strongly convex, surfaces smooth, shiny. Dorsal surface mostly black; elytral maculae pale yellow. Elytra widest near mid-length; humeri barely prominent; elytral punctuation dense and rather coarse. Each elytron with two large cloud-like maculae, their inner margin almost reaches the elytral margin, the outer margin far from the elytral suture.

***Measurements* (in mm)**. BL 5.3–5.9, EW 4.1–4.2.

#### Distribution.

Laos.

#### Remark.

Wang and Tomaszewska (2025) stated that Mr. Keita Matsumoto (NHMUK) provided them with photographs of a male “type” (with dissected aedeagus) (NHMUK 016412515) and a female “allotype” (NHMUK 016412518) for their study. Based on these images, they designated the male specimen as the lectotype of *Meilichius ornatus* Arrow, 1920. However, during examination of the type specimens of this species at the Natural History Museum, London, the first author of this paper discovered that the labels attached to the male and female specimens are the opposite of those cited in Wang and Tomaszewska (2025). This matter was verified with Mr. Keita Matsumoto, who confirmed that *Meilichius ornatus* is represented by two original type specimens: a female specimen labeled “type” and a male specimen labeled “co-type”. The label data for both specimens correspond exactly with the records made by the first author during his stay at the Natural History Museum. Mr. Matsumoto further explained that when he sent the photographs to Dr. Wang, a file name error inadvertently reversed the correspondence between the images and the specimens. Consequently, Dr. Wang, not having examined the type specimens in person, designated the lectotype and paralectotype based on this erroneous information. To rectify this error and ensure nomenclatural stability, we hereby re-designate the following specimens from the original syntype series:

- Lectotype (here designated): Male specimen, labeled “co-type”, collection number 016412518, deposited at the Natural History Museum, London.
- Paralectotype (here designated): Female specimen, labeled “type”, collection number 016412515, deposited at the Natural History Museum, London.

### Meilichius pachycerus

Taxon classificationAnimaliaColeopteraEndomychidae

Strohecker, 1951

B75CED1E-278A-50F9-BC9F-A6E172177242

Fig. 28

Meilichius pachycerus Strohecker, 1951: 166.

#### Type material.

***Holotype***: Borneo • sex undetermined; glued on a card with four labels as follows: “Mjoberg Collection” “W W Fnngs Bequest” “TYPE *Meilichius pachycerus* Strohecker” “California Academy of Sciences Type No. 8364”; (CAS).

#### Diagnosis.

Body short and ovate, strongly convex, surfaces smooth, shiny with metallic luster. Dorsal surface mostly ferruginous, antennomeres 1–5 yellowish, 6 infuscate at apex, 7–10 black, 11 black with its apical half yellow. Elytra widest near behind mid-length; humeri distinctly prominent; elytral punctuation sparse and coarse.

**Figure 28.**
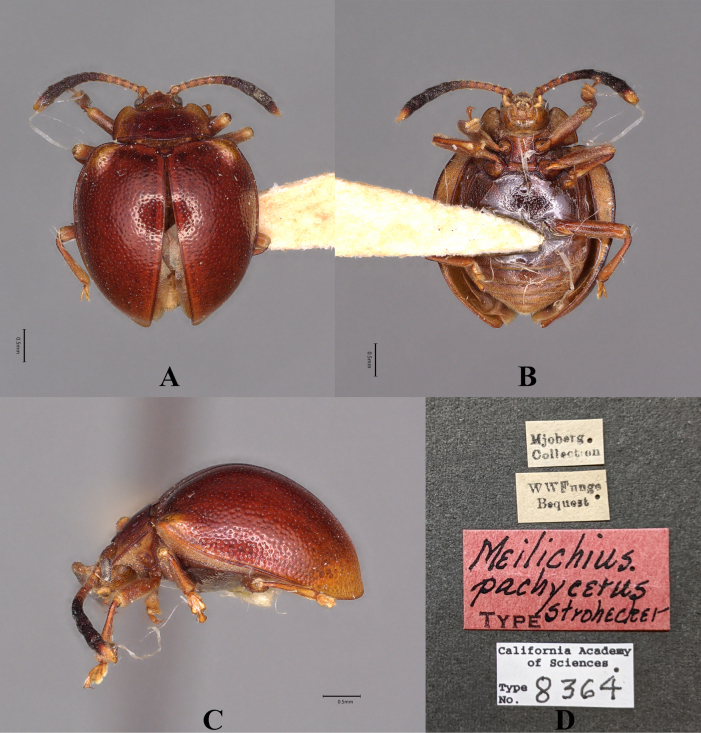
Holotype of *Meilichius pachycerus*. **A**. Dorsal view; **B**. Ventral view; **C**. Lateral view; **D**. Labels. Scale bars: 0.5 mm. (Photographed by Rachel Diaz-Bastin, CAS).

***Measurements* (in mm)**. BL 3.8.

#### Distribution.

Borneo.

#### Remark.

This species was examined in the present study with holotype photo observations, and the description and information presented here are based on Strohecker (1951).

### Meilichius politus

Taxon classificationAnimaliaColeopteraEndomychidae

Arrow, 1920

0B20E4A3-C95A-5D20-9CA9-9A0E6011EEFB

Fig. 29

Meilichius politus Arrow, 1920a: 71.

#### Type material examined.

***Holotype***: Sumatra • ♂; glued on a card with eight labels as follows: “Holo-type” “Type H. T.” “58140” “Sumatia Nias” “Yevman Mission” “Fry Coll. 1900 100.” “Meilichius politus. type arrow” “NHMUK 016510364”; (NHMUK).

#### Diagnosis.

Body short-ovate, strongly convex, surfaces smooth, shiny. Dorsal surface mostly bright reddish-brown, antennomeres 5–11 deep brown to black. Elytra widest near behind mid-length; humeri distinctly prominent; elytral punctuation sparse and strongly fine.

**Figure 29.**
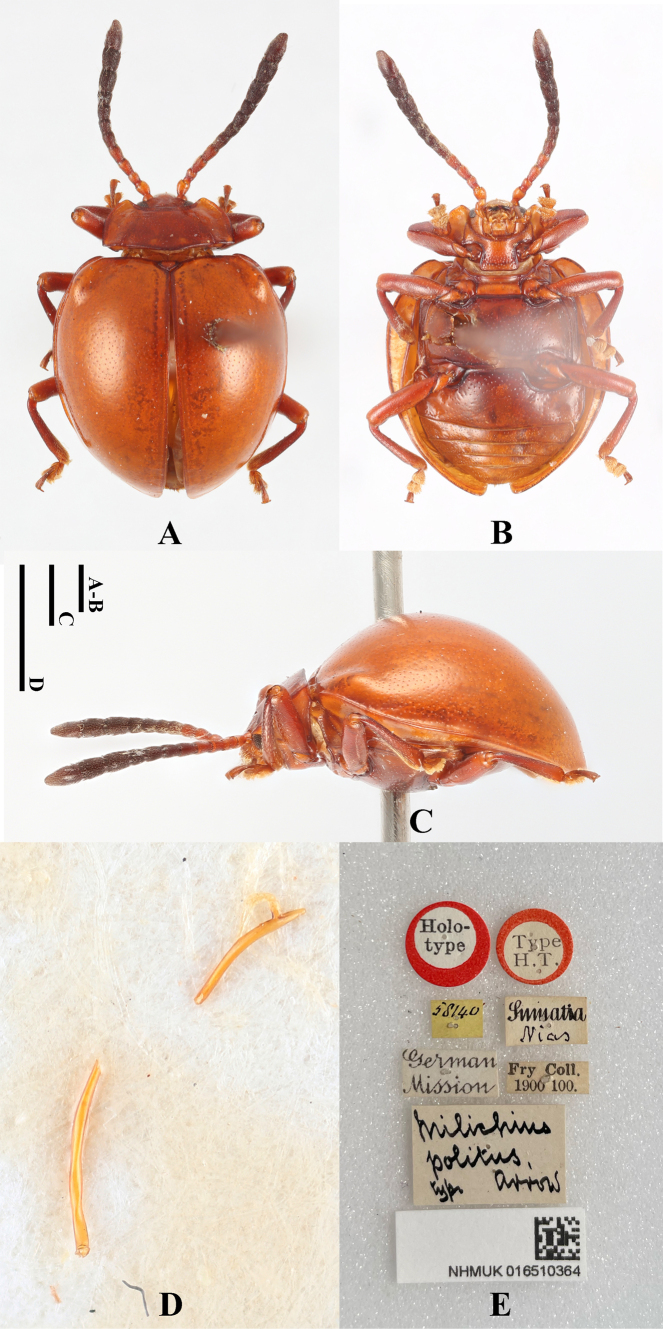
Holotype specimens of *Meilichius politus*. **A**. Dorsal view; **B**. Ventral view; **C**. Lateral view; **D**. Aedeagus; **E**. Labels. Scale bars: 1.0 mm.

***Measurements* (in mm)**. BL 4.4, EW 3.5.

#### Distribution.

Sumatra.

### Meilichius wukong

Taxon classificationAnimaliaColeopteraEndomychidae

Wang & Tomaszewska, 2025

6E058950-1833-572F-818B-283FEC0FD960

**Figure 30.**
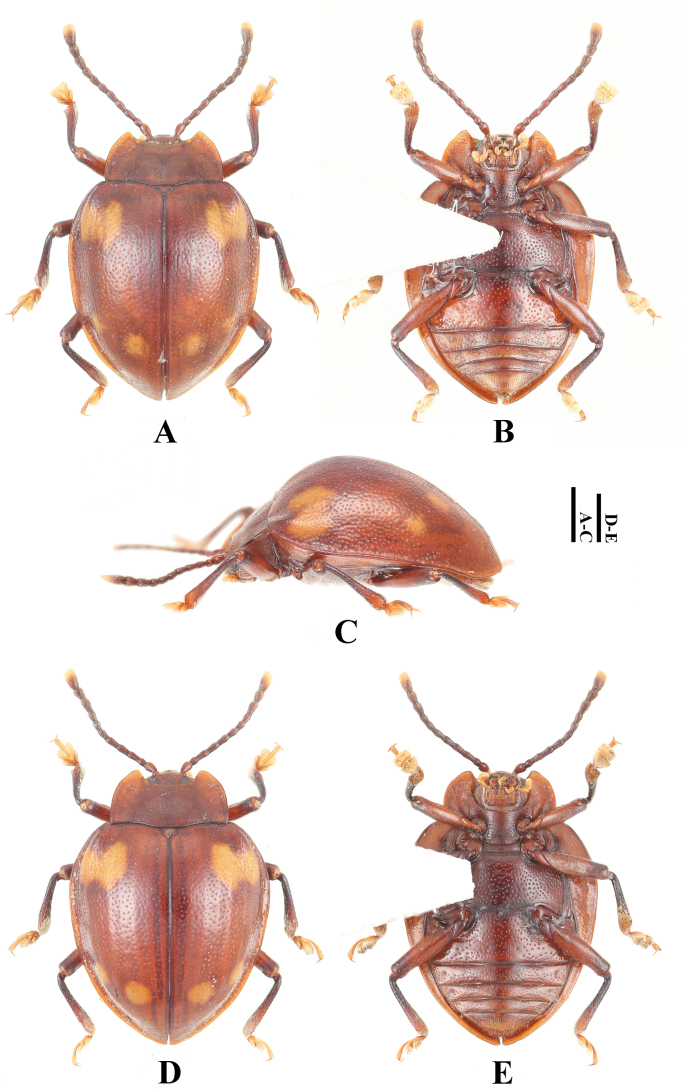
Habitus of *Meilichius wukong*. **A–C**. Male; **D, E**. Female: **A, D**. Dorsal view; **B, E**. Ventral view; **C**. Lateral view. Scale bars: 1.0 mm.

**Figure 31.**
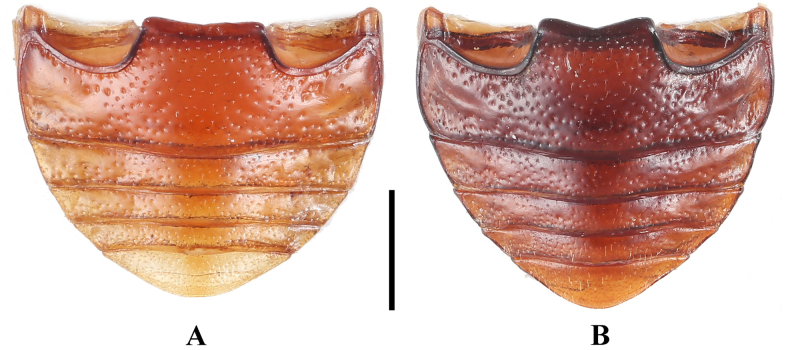
Abdomen of *Meilichius wukong*. (ventral view). **A**. Male; **B**. Female. Scale bar: 1.0 mm.

**Figure 32.**
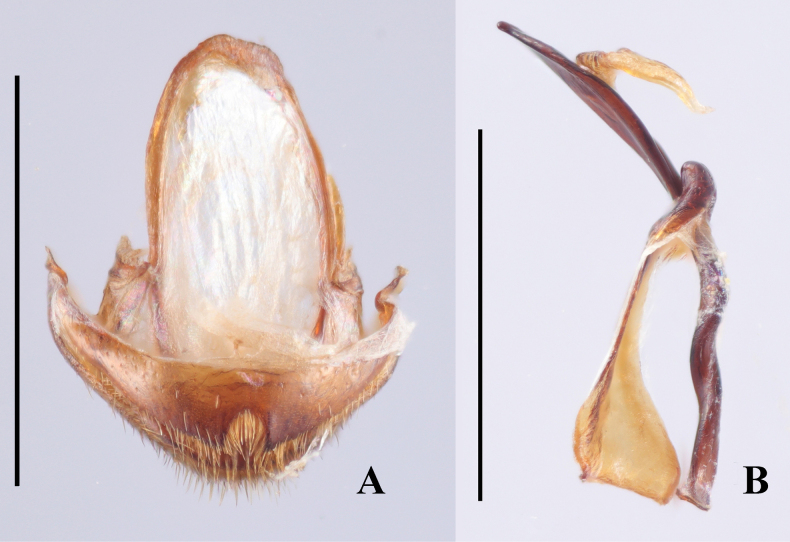
Male genital segment and Aedeagus of *Meilichius wukong*. **A**. Male genital segment and sternite VIII (ventrsal view); **B**. Aedeagus (lateral view). Scale bars: 1.0 mm.

**Figure 33.**
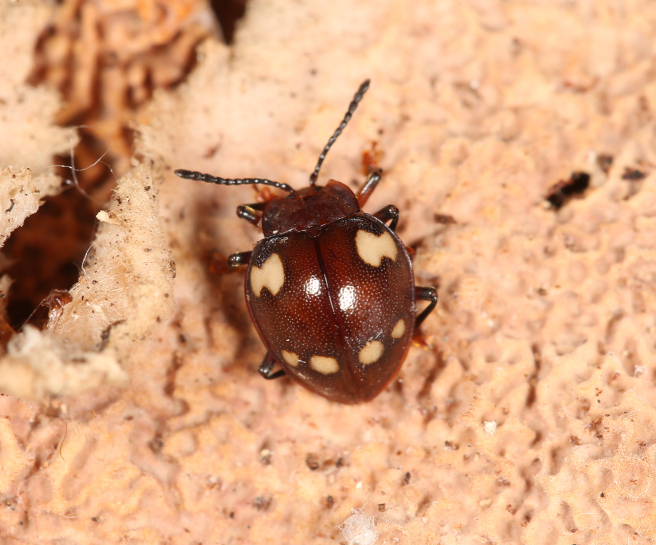
Live *Meilichius wukong*.

**Figure 34.**
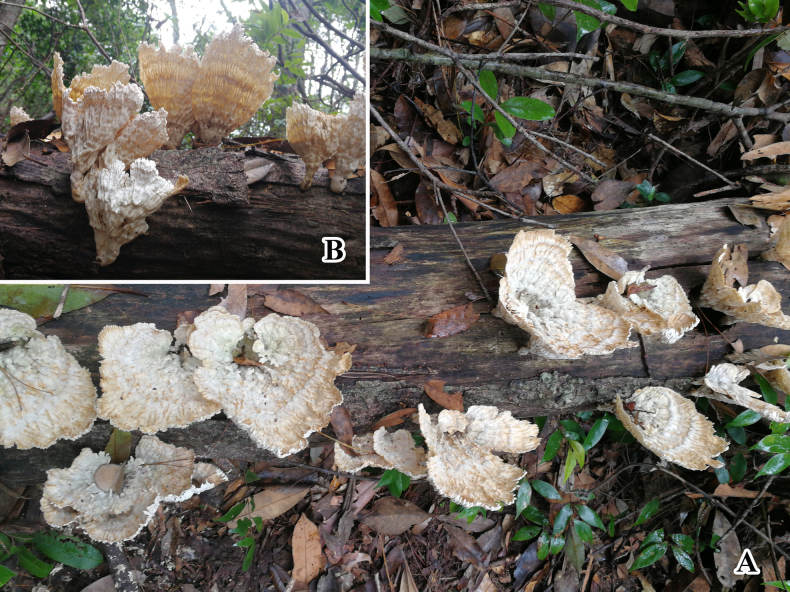
*Cymatoderma elegans* growing on a dead tree serves as an attractant for *Meilichius wukong*. **A**. Dorsal view; **B**. Lateral view.

Figs 30, 31, 32, 33, 34

Meilichius wukong Wang & Tomaszewska, 2025: 287.

#### Material examined.

China • 2♂♂, 3♀♀; **Guangxi**, Jinxiu County (金秀县), Changtong Town (长垌乡); 17.XI.2020; Chun-Fu Feng leg.; (NNHMC). China • 1♂; ditto except 3.III.2021; (NNHMC); China • 4♂♂2♀♀; ditto except 25.XII.2021; (NNHMC); China • 5♂♂10♀♀; ditto except 11.VIII.2022; (NNHMC); China • 1♀; **Guangdong**, Chebaling (车八岭), 24.715544°N, 114.154003°E; 764 m; 1.IV.2022; Pan-Pan Li leg.; (IZCAS). China • 1♀; ditto except 24.746948°N, 114.192158°E; 707 m; 16.VI.202; (IZCAS).

#### Diagnosis.

Body short-ovate, strongly convex, surfaces smooth, shiny. Dorsal surface mostly brown, antennomere 11 at apical 1/3, elytral maculae and tarsus pale yellow. Elytra widest near basal 1/4; humeri weakly prominent; elytral punctuation dense and coarse. Each elytron with three maculae, anterior maculae appearing as one large and one small oval maculae connected or narrowly separated; posterior maculae composed of two nearly round spots, obliquely arranged, subequal in size, and separated from each other.

***Measurements* (in mm)**. BL 4.6–5.4, BH 2.4–2.8, PL 1.4–1.8, PW 2.2–2.7, EL 4.2–4.6, EW 3.3–4.0, BH/BL 0.5, PL/PW 0.6–0.7, EL/EW 1.2–1.3, EL/PL 2.6–3.0, EW/PW 1.5.

#### Ecology.

The specimens of *Meilichius wukong* from Guangdong were collected by Mycophagy insects windows trap with fungus growing on a dead tree. The fungus was subsequently identified as *Cymatoderma elegans* (Fig. 34) based on Internal Transcribed Spacer (ITS) sequencing.

#### Remarks.

Upon examination of the specimens, we noted a sexual dimorphism in the shape of the ventrite 5 posterior margin: in the male (Fig. 31A) it is broadly rounded apically, whereas in the female (Fig. 31B) it is narrowly rounded. Additionally, in live or freshly collected specimens, the elytral maculae appear pale yellow and are encircled by a blackish band (Fig. 33). Upon drying, these maculae gradually turn orange-yellow, while the broad black band fades and becomes nearly invisible.

#### COI sequence.

COI sequence of *Meilichius wukong* resulted in a 681 bp. The GenBank accession number PX637245.

##### Key to the species of *Meilichius*

**Table:** 

1	Dorsal surfaces of elytra covered with short pubescence	***M. tomaszewskae* sp. nov**.
–	Dorsal surfaces of elytra smooth	**2**
2	Elytra without maculae	**3**
–	Elytra with maculae	**16**
3	Pronotum with lateral margins subparallel, from apical third distinctly converging toward the apex	**4**
–	Pronotum with lateral margins distinctly converging from the base toward the apex	**5**
4	Antennomeres 1–2 and 11 orange-yellow; 3–10 brown to brownish-black; humeral calli short, occupies basal ~1/6 of elytra	***M. ampliatus***
–	Antennomeres 1–5 ferruginous; 6–8 and 11 black; 9 and 10 pale yellow; humeral calli more longer, occupies basal ~1/5 of elytra	***M. impressicollis***
5	Body surfaces with distinctly metallic luster	**6**
–	Body surfaces without metallic luster	**8**
6	Body surfaces with aeneous luster	***M. aeneoniger***
–	Body surfaces with violet luster	**7**
7	Antennae almost brown; humeral calli orange	***M. ferrugineus***
–	Antennae mostly black, antennomeres 1–4 ferrugineous; humeral calli not orange	***M. expetitus***
8	Humeri weakly prominent	**9**
–	Humeri moderately to strongly prominent	**10**
9	Antennae almost uniformly brown to dark brown; elytral punctures coarse	***M. chebalingensis* sp. nov**.
–	Antennomeres 1–4 brown, antennomeres 5–10 and the basal half of the terminal one black; elytral punctures fine	***M. brevicollis***
10	Pronotum and elytra different colors	***M. nigricollis***
–	Pronotum and elytra concolorous	**11**
11	Elytra with humeral calli orange	***M. callosus***
–	Elytra with humeral calli not orange	**12**
12	Antennomeres 1–11 subconcolorous	***M. biplagiatus***
–	Antennomeres 1–11 distinctly bicolored	**13**
13	Body deep brown; elytral punctures distinctly coarse	**14**
–	Body deep pale brown; elytral punctures strongly fine	**15**
14	Antennomeres 1–4 and terminal one ferruginous; 5–10 black	***M. apicicornis***
–	Antennomeres 1–5 ferruginous, 6 darkened at apex, 7–10 black, and 11 black with the apical half yellow	***M. pachycerus***
15	Antennomeres 1–4 pale brown, 5–11 brown; legs concolorous with the pronotum. and elytra.	***M. politus***
–	Antennomeres 1–10 brown, terminal one pale brown; legs differently colored from pronotum and elytra	***M. fuscipes***
16	Elytral maculae distributed almost continuously from the base to the apex of the elytra, connected by bands of varying widths	***M. multimaculatus***
–	Elytral maculae not as above	**17**
17	Each elytron with one macula	**18**
–	Each elytron with more than one maculae	**20**
18	Elytra with strong metallic luster; elytral maculae transversely broad	***M. fasciatus***
–	Elytra without strong metallic luster; elytral maculae longitudinal half-oval shaped	**19**
19	Elytral maculae forming a single circular spot medially when elytra closed	***M. klapperichi***
–	Elytral maculae widely separates when elytra closed	***M. javanicus***
20	Each elytral with two maculae	**21**
–	Each elytral with three or four maculae	**23**
21	Body elongate oval; anterior and posterior elytral maculae with multiple U-shaped concavities and sharp teeth	***M. erotyloides***
–	Body short-ovate; anterior and posterior elytral maculae broad and large, approximately cloud-like shaped	**22**
22	Pronotum wine red, elytra black with copper-yellow luster; anterior and posterior elytral maculae connected by a broad band, or almost touching	***M. speciosus* sp. nov**.
–	Pronotum and elytra all black; elytra lacking copper-yellow luster; anterior and posterior elytral maculae widely separated	***M. ornatus***
23	Body approximately circular; each elytron with two pairs of oval, black spots, the anterior and posterior pairs widely separated	***M. geminatus***
–	Body short-ovate; each elytron with three maculae, anterior macula appearing as two oval maculae connected or narrowly separated; posterior macula composed of two round spots	***M. wukong***

## Supplementary Material

XML Treatment for Meilichius

XML Treatment for Meilichius chebalingensis

XML Treatment for Meilichius speciosus

XML Treatment for Meilichius tomaszewskae

XML Treatment for Meilichius aeneoniger

XML Treatment for Meilichius ampliatus

XML Treatment for Meilichius apicicornis

XML Treatment for Meilichius biplagiatus

XML Treatment for Meilichius brevicollis

XML Treatment for Meilichius callosus

XML Treatment for Meilichius erotyloides

XML Treatment for Meilichius expetitus

XML Treatment for Meilichius fasciatus

XML Treatment for Meilichius ferrugineus

XML Treatment for Meilichius fuscipes

XML Treatment for Meilichius geminatus

XML Treatment for Meilichius impressicollis

XML Treatment for Meilichius javanicus

XML Treatment for Meilichius klapperichi

XML Treatment for Meilichius multimaculatus

XML Treatment for Meilichius nigricollis

XML Treatment for Meilichius ornatus

XML Treatment for Meilichius pachycerus

XML Treatment for Meilichius politus

XML Treatment for Meilichius wukong

## Additional information

### Artificial Intelligence (AI) use

The authors accept full responsibility for the content of the manuscript, including the disclosure of any use of AI.

No AI tools were used in the preparation of this manuscript.

### Author ORCIDs

L.-X. Chang https://orcid.org/0009-0003-6174-1195

M. Bai https://orcid.org/0000-0001-9197-5900

W.-X. Bi https://orcid.org/0000-0003-1806-7679

### Data availability

All of the data that support the findings of this study are available in the main text.
